# AGEs promote the metastasis of colorectal cancer cells *via* centrosome amplification by KLF5–CEP57L1 axis

**DOI:** 10.1016/j.jbc.2025.111098

**Published:** 2025-12-22

**Authors:** Ji Zhong Zhao, Sheng Xian Fan, Jia Li Guo, Yu Cheng Lu, Xue Kai Bian, Ya Wen Han, Si Xian Xu, Meng Lu Zhao, Yuan Fei Li, Rong Peng Li, Shao Chin Lee

**Affiliations:** 1 Lab of Cell Biology, School of Life Sciences, Jiangsu Normal University, Xuzhou, Jiangsu, PR China; 2 Department of Colorectal Surgery, Nanjing Drum Tower Hospital, The Affiliated Hospital of Medical School, Nanjing University, Nanjing, China; 3 Biobank, Linyi People's Hospital, Linyi, Shandong, China; 4 Department of Oncology, The First Hospital, Shanxi Medical University, Taiyuan, Shanxi, China; 5 Medical School of Nantong University, Nantong, China

**Keywords:** AGEs, centrosome amplification, EMT, migration, invasion, metastasis, colorectal cancer, diabetes

## Abstract

Despite the accumulating evidence that diabetes and centrosome amplification (CA) are both associated with cancer cell metastasis, in particular the observations in gene-edited animal models, their relationships and the underlying molecular mechanisms remain unknown under pathophysiological conditions. In the present study, we examined if CA could serve as a biological link between diabetes and metastasis. Our results showed that, *in vitro*, advanced glycation end products (AGEs) promoted CA, migration, and invasion of HCT116 colorectal cancer cells, with the highest CA level in the migrated cell fraction, and upregulated FAM111B, which promoted epithelial–mesenchymal transition. Upon AGE treatment, Krüppel-like factor 5 (KLF5), Kelch-like (KLHL)13, and Cullin3 (CUL3) were downregulated and CEP57L1 was upregulated, respectively; the latter was due to an insufficient KLF5-mediated transcription of KLHL13 and CUL3 and therefore compromised protein ubiquitination degradation. Importantly, AGEs promoted CEP57L1-dependent metastasis of the cancer cells in a mouse model. In a cohort of cancer patients, KLF5, KLHL13, and CUL3 levels were lower, but CA and CEP57L1 were higher in cancer tissues, compared with noncancerous counterparts, which were more obvious in those with diabetes. Decreased KLF5, KLHL13, and CUL3, together, were associated with poorer survival. In conclusion, it is suggested that AGEs promote the cancer cell metastasis *via* CA by KLF5–CEP57L1 axis, which underlies diabetes-promoted cancer metastasis.

Diabetes is a common metabolic disease that increases the risk of cancer, including colorectal cancer (1). Accumulating evidence further suggests that it can promote tumor metastasis, which is linked to poor prognosis (2). Centrosome amplification (CA), a state in which a cell has more than two centrosomes, occurs commonly at the 3% to 15% in tumor cells, which can reach up to 28% in circulating tumor cells, which are the “seeds” of tumor metastasis (3). In clinical samples, CA level is associated with more advanced stages, metastases, and even recurrences of cancer. Notably, in gene-edited cell and animal models, CA has been directly demonstrated to induce tumorigenesis (4, 5) and increase the Rac1-mediated invasion of tumor cells (6), supporting that CA is a determining factor for tumor progression. We have reported that type 2 diabetes increases CA *in vivo* in peripheral mononuclear cells (7), with high glucose, insulin, free fatty acids (*i*.*e*., palmitic acid), and advanced glycation end products (AGEs) as inducers *via* various signaling pathways (6, 7). By the way, AGEs fall into a heterogeneous group of compounds irreversibly formed through nonenzymatic glycation and oxidation of proteins, lipids, and nucleic acids, a process markedly accelerated under diabetic hyperglycemia (8). Our results are consistent with those by Zhang *et al*. (9) that expression of the centriole protein PLK4, upregulation of which is commonly found to trigger CA, was increased in retinal cells of diabetic rats. Therefore, diabetes can induce CA, which was speculated as a biological link between diabetes and cancer initiation/metastasis (10).

Metastasis is regulated by a large number of factors that form a complex sequence of cell biology events, known as the invasion–metastasis cascade, which is composed of dissemination, dormancy, and colonization (11, 12). Epithelial–mesenchymal transition (EMT), characterized by multiple, dynamic transitions between epithelial and mesenchymal phenotypes, serves as a crucial driver of metastasis (13) and favors the invasion–metastasis cascade in all its stages (14); specifically, full and partial EMT lead to colonization failure and metastasis, respectively (11, 13, 14).

Krüppel-like factor 5 (KLF5) is a member of the KLF family, which is involved in multiple cellular processes, such as cell cycle, proliferation, stemness, and differentiation (15). The association between KLF5 and cancer is controversial, given that, depending on the experimental conditions, it can either promote cell migration and invasion (16) or inhibit cancer progression by preventing EMT (17). Similarly, E3 ligases can either promote or inhibit tumorigenesis or metastasis, depending on the adaptor (17, 18, 19). The cullin–RING E3 ligases (CRLs) are responsible for the ubiquitination of substrate proteins (20). CRL subunits include KLHL13, a member of the Kelch-like (KLHL) protein family, which is typically characterized by a BTB/poxvirus and zinc finger domain, a BACK domain, and a Kelch domain containing four to six Kelch repeat motifs (21). Cullin3 (CUL3) is a member of the seven-protein Cullin family. Finally, CEP57 and CEP57L1 are centrosomal proteins (CEPs) that take part in maintaining normal centrosome homeostasis (22). There is evidence that CEP57, which is highly expressed in prostate cancer, is linked to poor prognosis, as it contributes to oncogenic phenotype (23). CEP57L1, a CEP57 homolog, is not implicated in cancer. In the present study, we tested our hypothesis that AGEs promote the metastasis of colorectal cancer cells *via* CA mediated by specific signaling pathway(s).

## Results

### AGEs simultaneously promote CA and EMT in colorectal cancer cells

**Figure 1:**
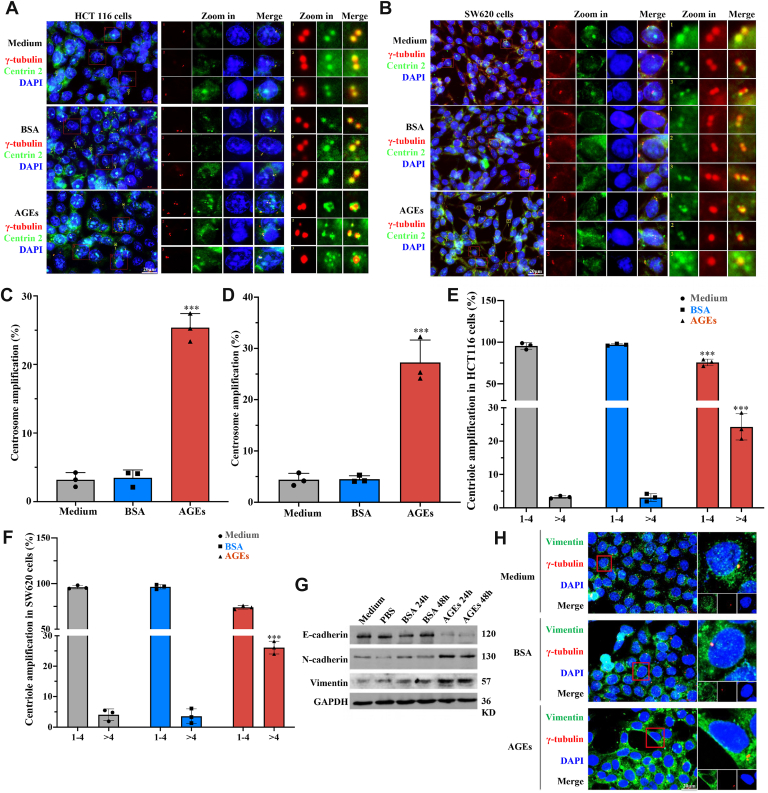
**AGEs simultaneously promote CA and EMT in colorectal cancer cells**. *A* and *B*, centrosomes were visualized using immunofluorescent staining of γ-tubulin (*red dots*) and centriole protein centrin 2 (*green dots*) in HCT116 (*A*) and SW620 (*B*) cells. *C* and *D*, AGEs significantly promoted CA both in HCT116 (*C*) and SW620 (*D*) cells by γ-tubulin staining. *E* and *F*, AGEs promoted centriole overduplication both in HCT116 (*E*) and SW620 (*F*) cells, as indicated by the presence of more than four centrioles in a cell. *G*, AGEs downregulated E-cadherin, whereas upregulated N-cadherin and vimentin. *H*, AGEs promoted the accumulation of vimentin to the border region of the cells. HCT116/SW620 cells were treated with 300 μg/ml AGEs for 48 h for centrosome staining, 36 h for Western blot analysis. ∗∗∗*p* < 0.001, compared with the medium and BSA groups; all assays were performed in triplicate. AGE, advanced glycation end product; BSA, bovine serum albumin; CA, centrosome amplification; EMT, epithelial–mesenchymal transition.

To verify that AGEs cause CA, we first confirmed their effective cellular uptake and stability by visualization of FITC-conjugated AGEs and minimal degradation data (<10% degradation at 48 h; Fig. S1, *A* and *B*). Consistent with our previous findings (24), AGEs induced CA in both HCT116 and SW620 cells; upon treatment, approximately 25% of the cells had CA indicated by γ-tubulin staining, and the centrioles were distributed around the centrosome, visualized by centrin 2 staining (Fig. 1, *A* and *B*). Time-course analysis revealed that CA occurred at 18 h post-treatment (Fig. S1*C*). Quantitatively, AGE increased the level of CA by more than sixfold (4% *versus* 25% in HCT116 cells and 4% *versus* 27% in SW620 cells; both *p* < 0.001) (Fig. 1, *C* and *D*). Centrin 2 staining showed that centriole overduplication (more than four centrin 2 staining dots in a cell) obviously occurred in both cell lines (3% *versus* 23% in HCT116 cells and 4% *versus* 26% in SW620 cells) (Fig. 1, *E* and *F*). AGEs also promoted EMT, as evidenced by decreased and increased expression levels of E-cadherin and N-cadherin–vimentin, respectively (Fig. 1*G* and S1*D*) and the accumulation of vimentin at the border region of treated cells (Fig. 1*H*). These results serve as evidence that diabetes favors metastasis of the colorectal cancer cells.

### AGEs promote *in vitro* migration and invasion of HCT116 cells *via* the CA–FAM111B–EMT route

**Figure 2:**
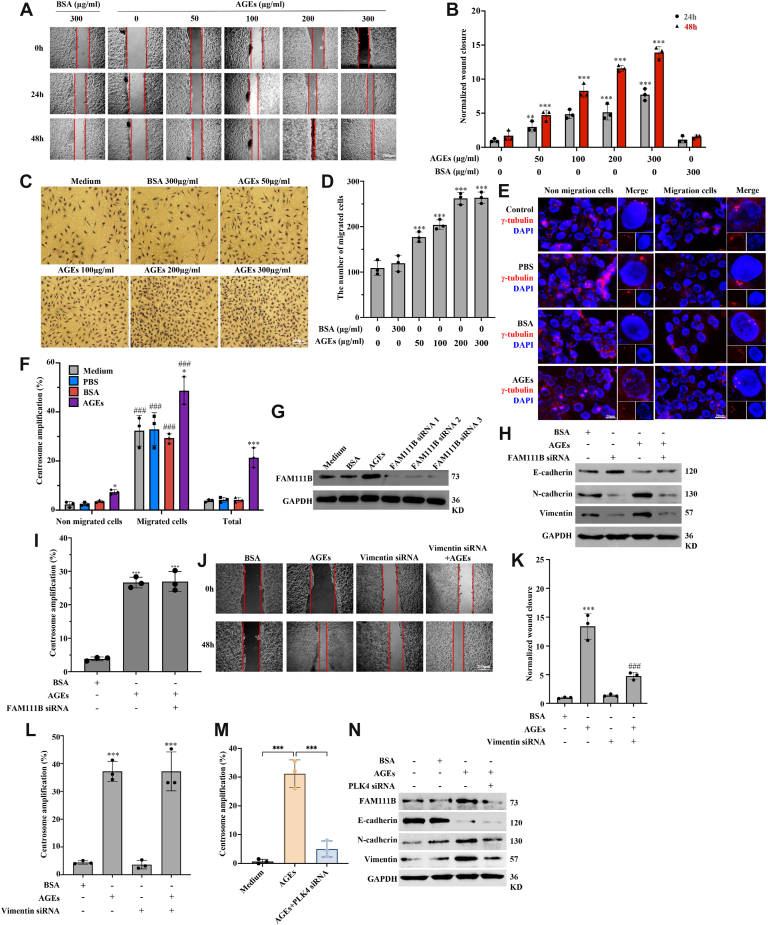
**AGEs promote *in vitro* migration and invasion of HCT116 cells *via* the CA–AM111B–EMT route**. *A*, AGEs promoted HCT116 cell migration in a concentration-dependent manner in the wound healing assay. AGEs (50–300 μM) were used for HCT116 treatment for 24 and 48 h. *B*, statistical data of migration shown in *A*. *C*, AGEs increased the migration in a concentration-dependent manner in the Transwell assay. *D*, the number of migrated cells in the Transwell experiments shown in *C*. *E*, the levels of CA in nonmigrated and migrated cells were calculated; centrosomes were visualized by the staining of γ-tubulin. *F*, CA was increased in the migrated cells, irrespective of the presence or the absence of AGEs. *G*, FAM111B was upregulated by AGEs, which could be effectively knocked down by siRNA species. *H*, FAM111B knockdown inhibited AGE-induced EMT and reversed the AGE-altered EMT molecular markers. *I*, FAM111B knockdown had no effect on the CA. *J*, vimentin knockdown inhibited the AGE-promoted migration. *K*, the statistical results of the migration. *L*, vimentin knockdown did not affect AGE-caused CA. *M*, PLK4 knockdown reduced AGE-induced CA. *N*, PLK4 knockdown attenuated AGE-induced upregulation of FAM111B and occurrence of EMT. HCT116/SW620 cells were treated with 300 μg/ml AGEs for 48 h for migration/invasion and 36 h for Western blot analysis. ∗∗*p* < 0.01 and ∗∗∗*p* < 0.001, compared with the medium and BSA groups; ##*p* < 0.01 and ###*p* < 0.001, compared with the AGE treatment group; all assays were performed in triplicate. AGE, advanced glycation end product; BSA, bovine serum albumin; CA, centrosome amplification; EMT, epithelial–mesenchymal transition.

We next examined the effects of AGEs on cancer cell migration and the molecular mechanisms. In wound healing (Fig. 2, *A* and *B*) and transwell (Fig. 2, *C* and *D*) assays, AGEs promoted migration and invasion in a dose-dependent manner. To investigate the correlation between CA and cell migration, we collected migrated cells on a cover slip underneath a transwell (Fig. S2*A*) to compare the CA levels in the migrated and nonmigrated cells. In the absence of AGEs, 30% of the migrated cells displayed CA, as compared with 5% in nonmigrated counterparts (*p* < 0.001), which was in comparison with 50% and 15%, respectively, in migrated and nonmigrated cells (*p* < 0.001) (Fig. 2, *E* and *F*). Interestingly, AGEs also upregulated EMT-associated protein FAM111B (25), which can localize in the centrosome (Fig. S2*B*). Knockdown of FAM111B by specific siRNAs (Fig. 2*G*) attenuated AGE-induced EMT but had no effect on CA (Fig. 2, *H* and *I*; Fig. S2, *C* and *D*). Furthermore, knockdown of vimentin (Fig. S2*E*) inhibited cell migration (Fig. 2, *J* and *K*) without affecting FAM111B and CA levels (Fig. S2*E* and 2*L*). Notably, knockdown of PLK4 (Fig. S2*F*), a key regulator of centrosome homeostasis, suppressed CA (Fig. 2*M*), thereby reversed the AGE-induced upregulation of FAM111B and the induction of EMT (Fig. 2*N* and S2*G*). These data support that AGEs favor metastasis and point to the underlying mechanistic pathway of CA–FAM111B–EMT.

### KLF5, KLHL13, CUL3, and CEP57L1 are CA-associated signaling molecules

**Figure 3:**
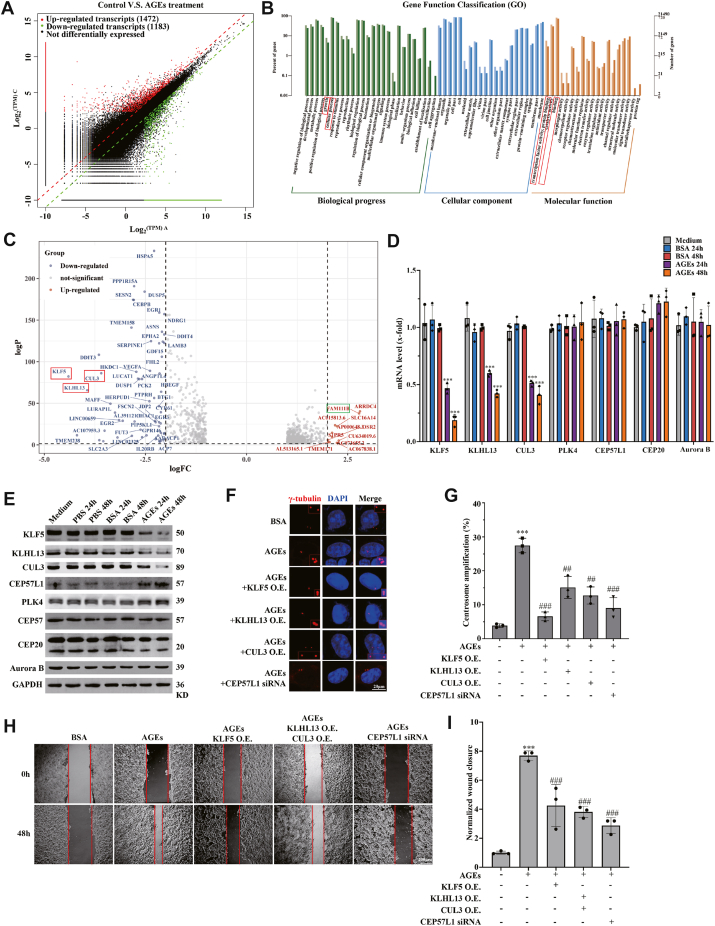
**KLF5, KLHL13, CUL3, and CEP57L1 are CA-associated signaling molecules**. *A*, transcriptomic profiling of the control and treated cells. *B*, the transcriptionally altered genes were clustered using GO annotation. *C*, the top of the downregulated or upregulated genes (50 for each) was listed. *D*, transcription level of the genes by quantitative PCR. AGEs inhibited the transcription of *KLF5*, *KLHL13*, and *CUL3* but not *PLK4*, *CEP57*, *CEP57L1*, *CEP20,* and *Aurora B*. *E*, AGEs decreased the protein level of KLF5, KLHL13, and CUL3, but not PLK4, CEP57, CEP20, and Aurora B, whereas they increased the protein level of CEP57L1. *F*, CA under overexpression of KLF5, KLHL13, or CUL3, and knockdown of CEP57L1; centrosomes were visualized by γ-tubulin staining. *G*, the statistical results of *F*; the experimental treatment inhibited the AGE-induced CA. *H*, the overexpression of KLF5 or KLHL13 + CUL3 and knockdown of CEP57L1 attenuated AGE-promoted cancer cell migration. *I*, the statistical results of the migration. HCT116/SW620 cells were treated with 300 μg/ml AGEs for 36 h for transcriptomics or Western blots and 48 h for migration–invasion analysis. ∗∗∗*p* < 0.001, compared with the medium and BSA groups. ##*p* < 0.01 and ^###^*p* < 0.001, compared with the AGE-treated group; all assays were performed in triplicate. AGE, advanced glycation end product; BSA, bovine serum albumin; CA, centrosome amplification; CUL3, Cullin3; GO, Gene Ontology; KLF5, Krüppel-like factor 5; KLHL, Kelch-like.

To further investigate molecules that may functionally regulate CA, which would provide tools to further assess the relationships among AGEs, CA, and cancer metastasis, we delineated the signaling pathways underlying CA. Transcriptomic profiling revealed that AGEs modulated the expression of 2655 genes (1472 downregulated and 1183 upregulated, respectively) (Fig. 3*A*). Gene Ontology enrichment showed that the differentially expressed genes were assigned to biological process, cell component, and molecular function categories in different terms related to cancer, such as cell growth and process, transcription factor activity, protein binding, and cell junction (Fig. 3*B*). Kyoto Encyclopedia of Genes and Genomes enrichment identified "Cell cycle" and "DNA replication" as the most significant pathways (Fig. S3*A*), which are closely related to centrosome homeostasis. Notably, *FAM111B* was among the 50 top upregulated genes (Fig. 3*C*), and *KLF5*, *KLHL13*, and *CUL3* were among the top downregulated genes. In the subsequent experiments, we evaluated the roles of KLF5, KLHL13, and CUL3 together with known CA signals, PLK4, CEP57, CEP57L1, CEP20, and Aurora B. The results confirmed that KLF5, KLHL13, and CUL3 were decreased at both mRNA and protein levels, whereas CEP57L1 was increased at the protein level only (Fig. 3, *D* and *E*; Fig. S3, *B*–*D*). Overexpression of KLF5, KLHL13, or CUL3 (Fig. S3, *E*–*G*) and knockdown of CEP57L1 (Fig. S3*H*) could not only inhibit CA (Fig. 3, *F* and *G*) but also reverse the AGE-promoted migration (Fig. 3 *I*, *H* and *I*). Thus, KLF5, KLHL13, CUL3, and CEP57L1 are signaling molecules that mediate the AGE-induced CA, which further supports that CA promotes metastasis.

### KLF5 activates the transcription of KLHL13 and CUL3 and inhibits CEP57L1 protein expression

**Figure 4:**
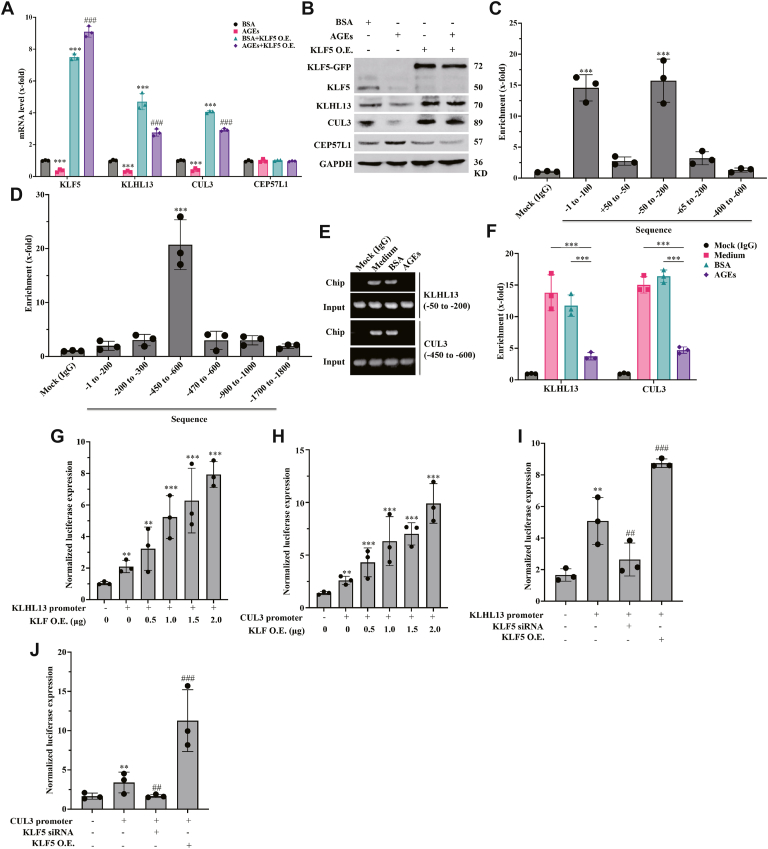
**KLF5 activates the transcription of KLHL13 and CUL3 and inhibits CEP57L1 protein expression**. *A*, overexpression of KLF5 counteracted the AGE-inhibited transcription of KLHL13 and CUL3 genes, without altering the transcription of CEP57L1. *B*, KLF5 overexpression increased protein levels of KLHL13 and CUL3 but inhibited CEP57L1. *C*, KLF5 bound to the −50 to −65 sequence in the promoter of KLHL13. *D*, KLF5 bound to the −450 to −470 sequence in the promoter of CUL3. *E* and *F*, AGE treatment attenuated the binding between KLF5 and the promoter of KLHL13 as well as CUL3, which was shown in the agarose gel electrophoresis (*E*) and quantitative PCR (*F*). *G* and *H*, the stepwise overexpression of KLF5 promoted expression of the reporter gene linked to the promoter of KLHL13 (*G*) or CUL3 (*H*). *I* and *J*, KLF5 knockdown inhibited the expression of reporter gene coupled to the promoter of KLHL13 (*I*) or CUL3 (*J*). HCT116/SW620 cells were treated with 300 μg/ml AGEs for 36 h for chromatin immunoprecipitation and Western blot analysis. ∗∗*p* < 0.01 and ∗∗∗*p* < 0.001, compared with the medium or BSA groups. ##*p* < 0.01 and ###*p* < 0.001, compared with the group using KLHL13 or CUL3 promoter; all assays were performed in triplicate. AGE, advanced glycation end product; BSA, bovine serum albumin; CEP, centrosomal protein; CUL3, Cullin3; KLF5, Krüppel-like factor 5; KLHL1, Krüppel-like factor 5.

Next, we investigated the specific roles of the signaling molecules and found that overexpression of KLF5 reversed the AGE-decreased mRNA and protein levels of KLHL13 and CUL3 and counteracted the decrease in protein level of CEP57L1 (Fig. 4, *A* and *B*; Fig. S4*A*). Based on the KLF5 binding sequence (Fig. S4*B*), the JASPAR tool identified multiple putative KLF5 binding sequences in the promoter regions of *KLHL13* (Table S1) and *CUL3* (Table S2). The top 10 candidate sequences were examined using the chromatin coimmunoprecipitation technique, which revealed that KLF5 bound to the sequences of −50 to −65 (including predicted sites 2 and 6) in the KLHL13 promoter (Fig. 4*C*) and −450 to −470 (including predicted sites 1 and 7) in the CUL3 promoter (Fig. 4*D*). The binding of endogenous KLF5 to these promoter sequences was abrogated by AGEs (Fig. 4, *E* and *F*). Furthermore, KLF5 overexpression increased the expression of reporter genes driven by KLHL13 or CUL3 promoter in a dose-dependent manner (Fig. 4, *G* and *H*), whereas knockdown of KLF5 had an inhibitory effect (Fig. 4, *I* and *J*). Thus, these findings suggest that KLF5 is a transcriptional activator of KLHL13 and CUL3 genes, which is inhibited by AGEs.

### A protein complex containing KLHL13 and CUL3 drives the ubiquitination and degradation of CEP57L1

**Figure 5:**
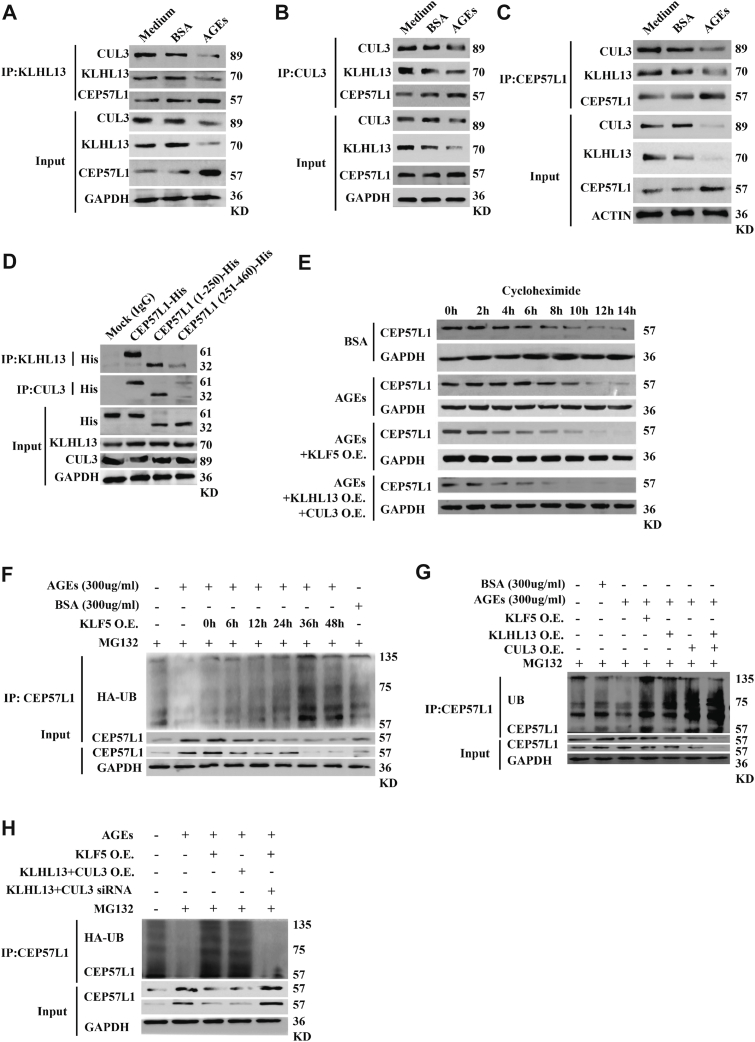
**A protein complex containing CUL3 and KLHL13 drives the ubiquitination and degradation of CEP57L1**. *A*–*C*, the interaction among KLHL13, CUL3, and CEP57L1 was evidenced by coimmunoprecipitation using antibodies against KLHL13 (*A*), CUL3 (*B*), or CEP57L1 (*C*). *D*, in the coimmunoprecipitation assay, KLHL13 or CUL3 predominantly interacted with the N-terminal region (1–250 residues) of CEP57L1. *E*, the half-life time of CEP57L1 protein was prolonged by AGEs but shortened by the overexpression of KLF5 or KLHL13 + CUL3. *F*, the ubiquitination of CEP57L1 was inhibited by AGEs but promoted by KLF5 overexpression in a time-dependent manner. *G*, the ubiquitination of CEP57L1 was increased by overexpression of KLF5, KLHL13, CUL3, or KLHL13 + CUL3. *H*, the increased ubiquitination of CEP57L1 by KLF5 overexpression was attenuated by simultaneous knockdown of KLHL13 and CUL3. MG132 (20 μM) was used to inhibit the activation of the proteasome. HCT116/SW620 cells were treated with 300 μg/ml AGEs for 36 h for Western blot analysis. All assays were performed in triplicate. AGE, advanced glycation end product; CEP, centrosomal protein; CUL3, Cullin3; KLHL, Kelch-like.

To understand better how AGEs upregulated CEP57L1, we consulted the STING database, which predicted that CUL3 might interact with several members of the KLHL superfamily, including KLHL13 but not CEP57L1 (Fig. S5*A*). Experimentally, antibodies against KLHL3, CUL3, or CEP57L1 were able to pull down each other, and their interactions were attenuated by AGEs (Fig. 5, *A*–*C*). Bioinformatic annotation also suggested that the N-terminal 1 to 250 amino acid residues of CEP57L1 might potentially bind to the Kelch domain of KLHL13 (Fig. S5*B*). Indeed, this N-terminal region of CEP57L1 was able to pull down KLHL13 and CUL3, whereas its C-terminal 251 to 460 region had minimal pull-down activity (Fig. 5*D*). These results suggest that KLHL13 and CUL3 function within a complex that binds to the CEP57L1 N-terminal region.

Moreover, we evaluated the half-life time of CEP57L1 in the presence of the translation inhibitor cycloheximide. AGEs prolonged the half-life time of CEP57L1, whereas its half-life was significantly shortened, when KLHL13 + CUL3 or their transcriptional activator KLF5 was overexpressed, even under AGE treatment (Fig. S5, *E* and *C*). In contrast, knockdown of either KLF5 or CUL3 extended the half-life of CEP57L1 (Fig. S5*D*). To determine whether AGEs increased the stability of CEP57L1 by reducing ubiquitination degradation, we performed pull-down assay with anti-CEP57L1 antibody, followed by Western blot analysis using antiubiquitin antibody. Indeed, AGEs attenuated CEP57L1 ubiquitination, which was reversed by overexpressing KLF5 in a time-dependent manner (Fig. 5*F* and S5*E*). On the other hand, CEP57L1 ubiquitination was enhanced by overexpression of KLHL13, CUL3, or KLHL13 + CUL3 (Figs. 5*H* and S5*G*). Notably, simultaneous knockdown of KLHL13 and CUL3 attenuated the effect of KLF5 overexpression on increasing CEP57L1 ubiquitination (Figs. 5*H* and S5*G*). Taken together, these results suggest that AGEs downregulate KLF5 and thus KLHL13–CUL3 complex, which compromises CEP57L1 ubiquitination degradation, and results in an increase in its protein level, leading eventually to CA.

### CEP57L1 contributes to centriole overduplication

**Figure 6:**
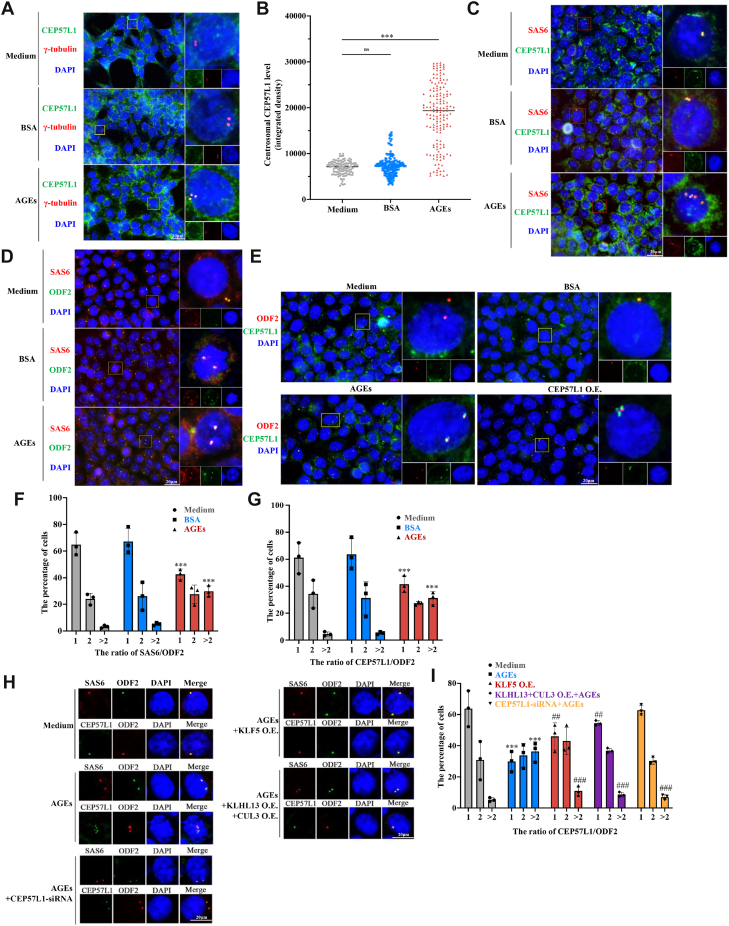
**CEP57L1 is localized at or near the cartwheel in centriole overduplication**. *A* and *B*, the localization of CEP57L1 on the centrosome, which was promoted by AGEs (*A*). The statistical data of *B*. *C*, colocalization of CEP57L1 (*green dots*) and SAS6 (*red dots*). *D*, the centrosomal localization of SAS6 (maker of cartwheel structure) and ODF2 (marker of mother centriole). *E*, both AGE treatment and CEP57L1 overexpression increased the distribution of CEP57L1 (*green dots*) on or around ODF2 (*red dots*). *F* and *G*, AGEs increased the ratios of SAS6/ODF2 and CEP57L1/ODF2. *H*, AGE-enhanced centrosomal localization of SAS6 and CEP57L1 was inhibited by CEP57L1 knockdown and the overexpression of KLF5 or KLHL13 + CUL3. *I*, AGE-increased ratios of SAS6/ODF2 and CEP57L1/ODF2 were inhibited when CEP57L1 was knocked down and KLF5 or KLHL13 + CUL3 was overexpressed. HCT116/SW620 cells were treated with 300 μg/ml AGEs for 48 h for centrosome staining. ∗∗∗*p* < 0.001, compared with the medium or BSA group. ##*p* < 0.01 and ###*p* < 0.001, compared with the AGE-treated group; all assays were performed in triplicate. AGE, advanced glycation end product; BSA, bovine serum albumin; CEP, centrosomal protein; CUL3, Cullin3; KLF5, Krüppel-like factor 5; KLHL, Kelch-like.

We were then interested in how CEP57L1 favored CA. Using immunofluorescent staining, we confirmed that CEP57L1 was localized on the centrosomes (Fig. 6*A*), which was increased by AGEs (Fig. 6*B*). Furthermore, CEP57L1 colocalized with SAS6 (Figs. 6*C* and S6), a key protein in the cartwheel structure that initiates centriole duplication. In approximately 20% of the AGE-treated cells, there were two or more fluorescent loci of SAS6 (Fig. 6*D*) or CEP57L1 (Fig. 6*E*), localized on or around a mother centriole indicated by positive ODF2 staining. Consequently, AGEs increased the SAS6-to-ODF2 and CEP57L1-to-ODF2 ratios by more than twofolds in approximately 32% of the cells (Fig. 6, *F* and *G*). Conversely, knockdown of CEP57L1 or overexpression of KLF5–KLHL13 + CUL3 substantially lowered the ratios (Fig. 6, *H* and *I*). These data suggest that CEP57L1 acts on or near the cartwheel structure to cause ectopic procentriole formation (26) close to mother centrioles.

### AGEs promote metastasis of colorectal cancer cells *via* CA in a xenograft mouse model

**Figure 7:**
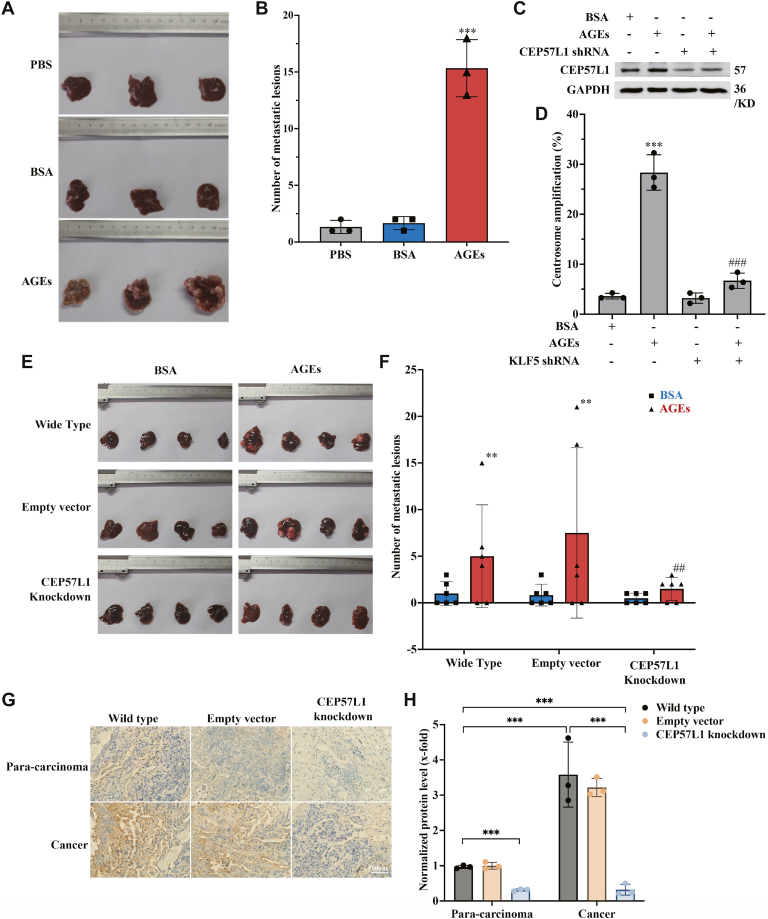
**AGEs promote metastasis of colorectal cancer cells *via* CA in a xenograft mouse model**. *A* and *B*, AGEs promoted the liver metastasis of the cancer cells in the nude mice model. *C*, stable knockdown of CEP57L1 in the HCT116 cells, even in the AGE-treated cells. *D*, stable knockdown of CEP57L1 significantly inhibited the AGE-induced CA. *E*, AGEs promoted the liver metastasis of the wildtype cancer cells. The AGE-triggered metastasis was completely inhibited when CEP57L1 was knocked down. *F*, the statistics of the metastatic lesions in different experimental groups as shown in *E*. *G*, the AGE-increased CEP57L1 level (*brown color*) in the metastatic tumor tissues was completely inhibited by CEP57L1 knockdown. *H*, the expression level of CEP57L1 was normalized and quantified using the staining intensity score. ∗∗*p* < 0.01 and ∗∗∗*p* < 0.001, compared with the wildtype or vector-only cells. ###*p* < 0.001, compared with the AGE-treated group; all assays were performed in triplicate. AGE, advanced glycation end product; CA, centrosome amplification; CEP, centrosomal protein.

Next, we moved to test whether AGEs could promote cancer cell metastasis *via* CA in the mouse model (Fig. S7*A*), for which we compared the metastasis potential of wildtype cells and those with knockdown of CEP57L1 (CA inhibition). Our results confirmed that i.p. injection of AGEs increased the liver metastasis of the wildtype cancer cells that were inoculated into the splenic tips of nude mice (Fig. 7, *A* and *B*). Then, we generated two cell lines, with stable knockdown of CEP57L1 or an empty vector for the experiments. The stable knockdown of CEP57L1 (Figs. 7*C* and S7*B*) *in vitro* inhibited the CA (Fig. 7*D*). The AGE-increased liver metastasis was also inhibited *in vivo* when the CEP57L1 knockdown cells, but not those cells transfected with vector alone, were used in the animal study, as compared with the metastasis experiments that used wildtype cells (Fig. 7*E*). Specifically, the number of metastatic lesions in the liver was 2.5 (wildtype), 2 (vector alone), and 1 (CEP57L1 knockdown) without AGE injection, which was increased to 10, 12, and 2.5, respectively, upon the injection of AGEs (Fig. 7*F*). Furthermore, a strong positive correlation was observed between metastatic burden and CA frequency within the lesions: CA frequencies were <1% in lesion-free livers, ∼10% in livers with 2 to 5 nodules, ∼18% with 6 to 10 nodules, and ∼30% with over 10 nodules (Fig. S7*C*). Consistent with the functional role of CEP57L1, its expression was higher in cancer tissues than paracarcinoma counterparts, irrespective of cell type (Fig. 7, *G* and *H*). The level of CEP57L1 in tumor and paracarcinoma tissues in mice inoculated with CEP57L1 knockdown cells without AGE treatment was not quantified because of the shortage of metastatic tissues. These results suggest that AGEs induce metastasis *via* CA by pathophysiological factors rather than gene editing, which can potentially produce biased interpretations.

### CA is upregulated in diabetes, and KLF5, KLHL13, and CUL3 levels are associated with the survival of colorectal cancer patients

**Figure 8:**
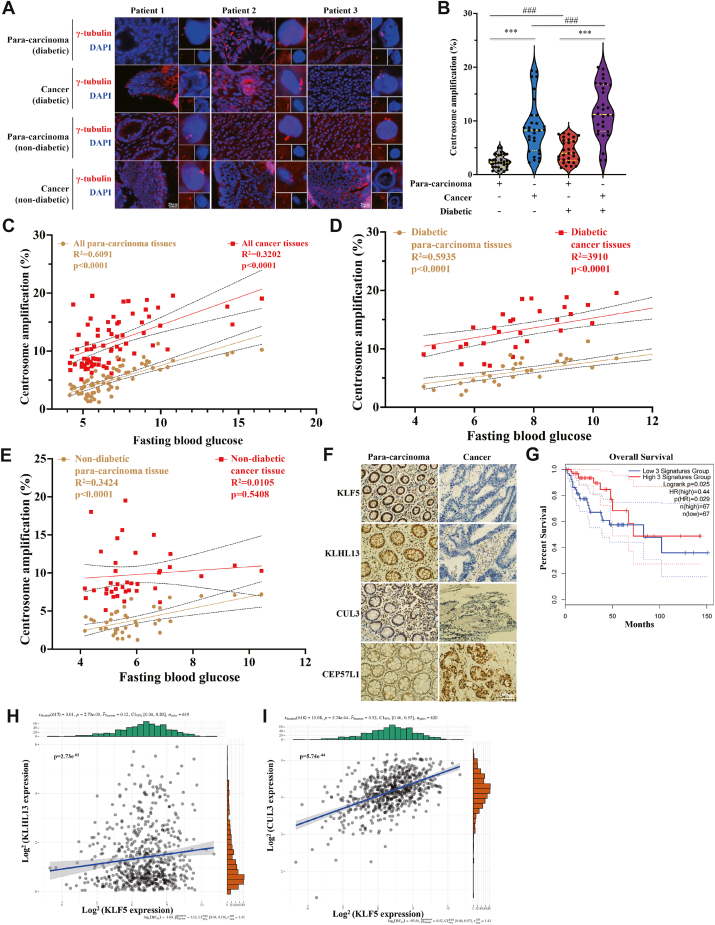
**CA is upregulated in diabetes, and the expression levels of KLF5, KLHL13, and CUL3 are associated with the survival of colorectal cancer patients**. *A*, CA was visualized using immunofluorescence staining of γ-tubulin in paracarcinoma and cancer tissues from diabetic or nondiabetic colorectal cancer patients. *B*, statistics of *A*. CA was higher in both paracarcinoma and cancer tissues from diabetic patients than in the tissues from the nondiabetic subjects. *C*–*E*, the correlation between CA and fasting blood glucose in all patients (*C*), and those with (*D*) or without (*E*) diabetes. *F*, the expression of KLF5, KLHL13, or CUL3 was higher in the paracarcinoma samples than in the cancer tissues. Notably, the cancer tissues had an increased level of CEP57L1, compared with the paracarcinoma samples. *G*, the expression level of KLF5, KLHL13, and CUL3, together as a parameter, was associated with the overall survival of intestinal cancer patients. *H* and *I*, the correlation between the transcription of KLF5 and KLHL13 (*H*) or CUL3 (*I*). ∗∗∗*p* < 0.001, compared with the paracarcinoma tissues; ###*p* < 0.001, compared with the nondiabetic subjects; all assays were performed in triplicate. CA, centrosome amplification; CUL3, Cullin3; KLF5, Krüppel-like factor 5; KLHL, Kelch-like.

Finally, we attempted to seek evidence for a role of CA in metastasis of colorectal cancer among patients with (n = 106) or without (n = 106) diabetes (Table S3) and found that CA levels were 8% and 13% in colorectal tissues (*p* < 0.05), as compared with 2.5% and 6% in paracarcinoma tissues from patients without and with diabetes, respectively (*p* < 0.01) (Fig. 8, *A* and *B*). Moreover, CA levels were positively correlated with fasting blood glucose in both paracarcinoma and cancerous tissues. This relationship was evident in all colorectal cancer patients (Fig. 8*C*) and held true for the subgroup with diabetes (Fig. 8*D*), with both correlations being statistically significant (*p* < 0.001). In patients without diabetes, there was a positive correlation in paracarcinoma tissues (*p* < 0.001) but not cancer tissues (*p* > 0.01) (Fig. 8*E*).

For insights into the association between the signaling molecules and prognosis, we quantified the expression of KLF5, KLHL13, CUL3, and CEP57L1 proteins. Cancer tissues had lower levels of KLF5, KLHL13, and CUL3 but higher levels of CEP57L1 than paracarcinoma counterparts (Figs. 8*F* and S8*A*), which was exaggerated by the presence of diabetes (Fig. S8, *B* and *C*). KLF5 expression correlated significantly with the levels of KLHL13 and CUL3 (Fig. 8, *H*–*I*), which was consistent with our *in vitro* results. According to The Cancer Genome Atlas (TCGA) database, the decreased expression of *KLF5*, *KLHL13*, and *CUL3* in colorectal cancer tissues (Fig. S8, *D*–*F*) was predictive of poorer overall survival (Fig. 8*G*). The expression of *CEP57L1* was statistically higher in cancer patients, and it did exhibit a negative correlation with the overall survival of cancer patients ((Fig. S8, *G* and *H*). In addition, decreased expression of *KLF5* (cancer stage II), *KLHL13* (stages III and IV), and *CUL3* (stages II and IV) correlated with advanced tumor staging. Whereas, a consistent increase in *CEP57L1* mRNA level was seen across stages (Fig. S8, *I*–*L*). Collectively, these results are consistent with our view that CA favors the metastasis of colorectal cancer *via* the KLF5–KLHL13–CUL13 signaling pathway, which results in insufficient degradation and therefore increment in CEP57L1.

## Discussion

**Figure 9:**
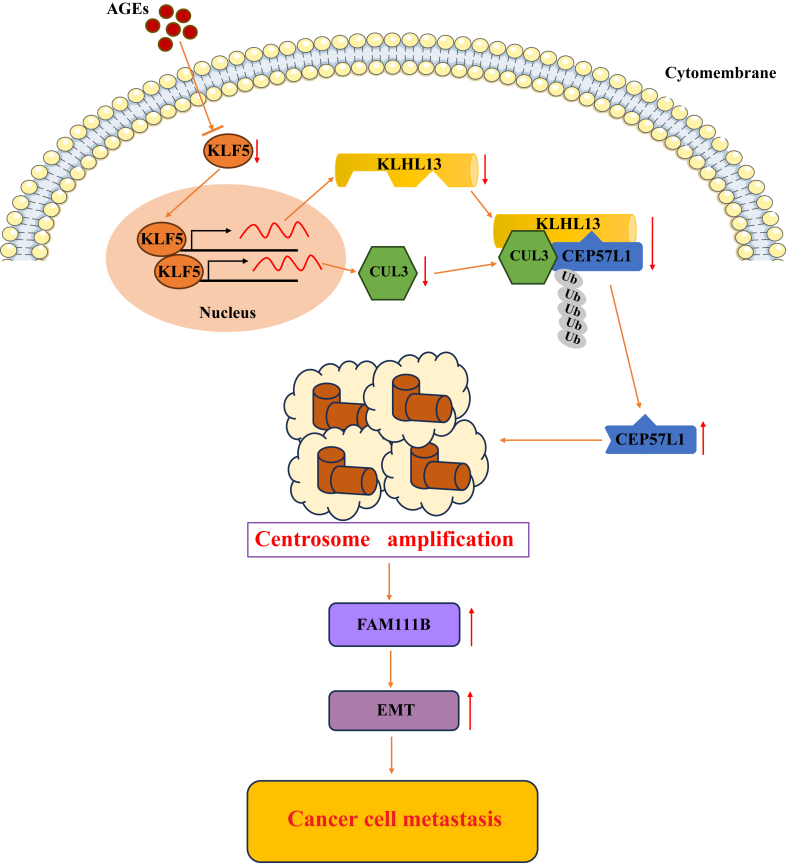
**Proposed model for AGE-promoted colorectal cancer metastasis *via* the KLF5–CEP57L1–centrosome amplification (CA) axis**. AGEs downregulate the transcription factor KLF5, which in turn suppresses expression of the E3 ubiquitin ligase components KLHL13 and CUL3. The impaired KLHL13–CUL3 complex fails to ubiquitinate CEP57L1, leading to its stabilization and accumulation. Elevated CEP57L1 induces CA, which enhances cellular invasion and cancer metastasis. AGE, advanced glycation end product; CEP, centrosomal protein; CUL3, Cullin3; KLF5, Krüppel-like factor 5; KLHL, Kelch-like.

The impressive evidence for CA in metastasis comes from studies using gene-edited models. However, results from editing a single gene leave it ambiguous whether the metastatic phenotype is a direct consequence of CA itself or an independent effect of the altered gene, potentially leading to misleading interpretations. More direct evidence obtained under conditions that closely mimic the pathophysiological microenvironment is therefore required to establish CA as a driver of metastasis. Indeed, in our study, using CA induced by pathophysiological factors in diabetes as an experimental model, we demonstrate that AGEs promote CA in colorectal cancer cells, which in turn enhances EMT, migration, and invasion *in vitro*, and favors metastasis *in vivo*, with a clearly delineated molecular mechanism of the AGE–KLF5–CEP57L1 axis (Fig. 9). These results project a compelling picture that CA acts as a trigger for metastasis, at least in the context of diabetes, thereby claiming that CA is a critical biological link between diabetes and cancer metastasis.

At the molecular level, it is clear that AGEs lowered the expression of KLF5, KLHL13, and CUL3 (Fig. 3, *D* and *E*). KLF5, a Kruppel-like transcription factor implicated in migration, invasion, and EMT (27, 28), and transcriptionally regulates KLHL13 and CUL3, which are components of the CRL E3 ubiquitin ligase complex (29). The reduction in KLF5 results in compromised expression of KLHL13 and CUL3, thereby disrupting the integrity and function of this ubiquitination complex. We had not probed into the signaling molecules upstream of KLF5 but suspect that the receptor for advanced glycation end product/mitogen-activated protein kinase/NF-κB is involved, as the receptor for advanced glycation end product is known as the receptor of AGEs (30). It should be mentioned that AGEs can promote cancer cell metastasis *via* SP1 (31), which is a transcriptional activator of KLF5 (32). Since those authors did not provide data on KLF5, we are unable to compare the outcomes of the two studies.

Importantly, AGEs upregulated the protein, but not mRNA, level of CEP57L1, a centrosomal component (22). We demonstrated that this increase is due to insufficient ubiquitination degradation by the KLHL13–CUL3 E3 ligase complex, which consequently causes CA, as evidenced by four key lines of evidence: (1) AGEs prolonged the half-life of CEP57L1, an effect reversed by overexpressing KLF5 or KLHL13 + CUL3; (2) AGEs attenuated CEP57L1 ubiquitination, which was enhanced by KLF5, KLHL13, or CUL3 overexpression; (3) the N-terminal domain of CEP57L1 physically interacts with the KLHL13–CUL3 complex; and (4) knockdown of CEP57L1 was sufficient to decrease CA levels. These findings establish a definitive mechanism whereby AGEs decrease KLF5 expression to downregulate the KLHL13–CUL3 complex, leading to reduced ubiquitination and degradation of CEP57L1, resulting in its accumulation. The subsequent increase in CEP57L1 stability drives CA, thereby positioning the KLF5–KLHL13–CUL3–CEP57L1 axis as a core mechanistic pathway through which AGEs modulate CA to enhance metastatic potential.

CEP57L1 colocalizes with SAS6, a key component of the cartwheel structure, at the centrosome (Fig. 6*C*). Although high-resolution imaging for localizing CEP57L1 within the cartwheel itself was not produced because of technical difficulty, it clearly shows that colocalization and binding with SAS6 (Fig. S6) strongly support our central hypothesis: in the pathological context of colorectal cancer cells under diabetic-like conditions, the accumulation of CEP57L1 protein, resulting from impaired KLHL13–CUL3-mediated ubiquitination, facilitates centriole overduplication by promoting the ectopic recruitment and stabilization of the cartwheel assembly machinery, specifically SAS6, at the mother centriole. This hypothesis is grounded in our direct observations that CEP57L1 knockdown, as well as the upstream intervention of KLF5 overexpression, effectively blocks AGE-mediated SAS6 accumulation and subsequent CA (Fig. 6*H*). This proposed that the gain-of-function mechanism for CEP57L1 overexpression differs from its reported role in maintaining centriole cohesion in noncancerous models (22, 33); this critical discrepancy underscores that the functional consequences of altering CEPs are highly context dependent, with pre-existing oncogenic stress potentially unveiling a distinct, pathological function. Importantly, our data reveal that the AGE–KLF5–KLHL13–CUL3 axis specifically regulates CEP57L1 without affecting its paralog CEP57 (Figs. 3*E* and S2*A*), highlighting a nonredundant and pathway-specific role for CEP57L1 in this diabetic cancer context. While our model focuses on CEP57L1's interaction with the cartwheel apparatus, it is situated within a broader network of CA-regulating pathways, including the established KLHL21–CUL3–Aurora B axis (34) and other factors like SP1 (31) and Rac1 (6) implicated in AGE signaling, collectively indicating that multiple molecular inputs can converge on the centrosome to drive amplification under diabetic conditions.

Our *in vivo* results provide compelling evidence for a causal role of CA in promoting metastasis, which is substantiated by two critical lines of experimentation. First, we established that the pathophysiological trigger AGEs promote metastasis in a CA-dependent manner. This was demonstrated by combining the administration of AGEs with the use of genetically engineered cells: whereas AGEs significantly increased liver metastasis of wildtype cancer cells, this prometastatic effect was virtually abolished when CEP57L1-knockdown cells, which are resistant to AGE-induced CA, were inoculated. This key genetic intervention provides direct evidence that the ability of AGEs to promote metastasis is contingent upon their capacity to induce CA. Second, if the pathway of diabetes-promoting metastasis *via* CA holds clinical relevance, patient data should reflect the correlations between hyperglycemia, CA, and metastatic progression. Indeed, our analysis of clinical samples confirmed this relationship, revealing that diabetic colorectal cancer patients exhibited significantly higher levels of CA in both cancerous and paracarcinoma tissues compared with their nondiabetic counterparts, with CA levels positively correlating with fasting blood glucose. Furthermore, the expression pattern of the key signaling molecules, downregulation of KLF5, KLHL13, and CUL3, alongside upregulation of CEP57L1, in patient tumors aligns perfectly with our proposed molecular axis. Collectively, the convergence of controlled animal studies and human clinical data strongly positions CA as a critical biological link between diabetes and cancer metastasis.

We have noticed that CA can occur without diabetes *via* endogenous/exogenous physical, chemical, and biological factors/agents. Specifically, reactive oxygen species and DNA damage, which are frequent events in humans as well as oncogenic viral infection, are capable of inducing CA (10). In fact, reactive oxygen species production and DNA damage occur at higher levels in cancer than in noncancer tissues (35). Therefore, CA might be a universal prometastasis mechanism, with or without the presence of diabetes.

In summary, this study delineates a complete signaling pathway from diabetic stimuli to metastatic progression: AGEs downregulate transcription factor KLF5, impairing the KLHL13–CUL3 E3 ligase complex because of their compromised expressions by KLF5, which leads to the reduction of ubiquitination degradation by KLHL13–CUL3 and therefore stabilization of the CEP57L1. The resultant CA drives enhanced invasiveness *in vitro* and liver metastasis *in vivo*. The clinical relevance of this axis is confirmed by the inverse correlation between KLF5–KLHL13–CUL3 expression and patient survival, firmly establishing CA as a critical mechanistic link between diabetes and colorectal cancer metastasis (Fig. 9).

## Experimental procedures

### Cells and antibodies

The HCT116 and SW620 colorectal cancer cells were obtained from the National Collection of Authenticated Cell Cultures. Antibodies against CEP57 (catalog no.: D224518; rabbit), KLHL13 (catalog no.: D194772; mouse), CUL3 (catalog no.: D290363, rabbit), and PLK4 (catalog no.: D222889, rabbit) were purchased from Sangon Biotech. Those against CEP57L1 (catalog no.: 24957-1-AP, rabbit), Aurora B (catalog no.: 39262, rabbit), CEP20 (catalog no.: 26409-1-AP, rabbit), and GAPDH (catalog no.: 67763-1-Ig, mouse) were from Proteintech. Secondary antibodies, goat anti-rabbit (catalog no.: SA00001-2) and anti-mouse (catalog no.: SA00001-1), were both from Proteintech. The Alexa Fluor 488–conjugated goat anti-rabbit secondary antibody and Alexa Fluor 594–conjugated goat anti-mouse secondary antibodies were purchased from Abways.

### The preparation of AGEs and FITC–AGE

Bovine serum albumin (BSA) at the concentration of 50 mg/ml (Sigma–Aldrich) was incubated with 0.5 mmol/l d-glucose (Absin) in 0.2 M PBS (pH 7.4) for 8 weeks under sterile conditions at 37 °C in the dark to prevent photodegradation. Following incubation, unincorporated glucose and other low-molecular-weight reactants were removed *via* extensive dialysis against PBS (0.2 mmol/l, pH 7.4) using a dialysis membrane with a 12 to 14 kDa molecular weight cutoff, with multiple buffer changes over 48 h. A control BSA solution was prepared in parallel without d-glucose. The quality was assured by characterizing their specific fluorescent properties using a fluorospectrophotometer (SpectraMax M5) with excitation and emission wavelengths set at 370 nm and 440 nm, respectively (36).

Labeling of AGEs with FITC was performed by conjugating FITC isomer I (Sigma–Aldrich) to protein in a 0.1 M carbonate buffer (pH 9.0) at a 10:1 M ratio (FITC:AGEs). Briefly, a 10 mg/ml AGE solution was incubated with a freshly prepared 1 mg/ml FITC solution in anhydrous dimethyl sulfoxide for 8 h at 4 °C in the dark with gentle agitation. The reaction was quenched with 50 mM NH_4_Cl for 1 h. Unconjugated FITC was removed by extensive dialysis against PBS (pH 7.4) using a 10 kDa molecular weight cutoff membrane, with multiple buffer changes until the dialysate was devoid of fluorescence. The FITC–AGE conjugate was characterized spectrophotometrically by measuring absorbance at 280 nm and 495 nm to determine protein concentration and the fluorophore-to-protein ratio, aliquoted, and stored at −20 °C under light protection until use.

### Cell culture and treatment

Both HCT116 and SW620 cells were cultured in Dulbecco's modified Eagle's medium (Lonza) supplemented with 10% (v/v) fetal bovine serum (FBS; Gibco), 50 U/ml penicillin, and 50 μg/ml streptomycin (Sigma–Aldrich). For the experimental treatments, AGEs (300 μg/ml) were added directly to the cell culture medium. Samples treated for 48 h were used for the staining of centrosomes and migration–invasion assays. Those treated for 36 h were used for transcriptomic profiling and Western blot analysis.

### Immunofluorescent staining of proteins

Approximately 50,000 cells were seeded onto a cover slip in 6-well plate for treatment after attachment. The cells were first fixed in a 1:1 mixture of cold methanol and acetone (6 min, −20 °C), followed by a wash with PBS and then incubated with 0.1% Triton X-100 for 15 min and 3% BSA for 1 h. Following the incubation with a primary antibody in 3% BSA in PBS overnight at 4 °C, cells were washed twice with PBS and incubated with an Alexa Fluor 488– or 549–conjugated secondary antibody in 3% BSA in 1× PBS for 1 h at room temperature in the dark. After washing (PBS, 3 × 5 min), cells were mounted with mounting medium and photographed under a Leica biological microscope DM6000B (Leica) with a 1.4 numerical aperture oil-immersion lens. Images were then analyzed using ImageJ software (National Institutes of Health) to count the number of centrosomes. To calculate the percentage of the cells with CA, 200 cells were counted, and the CA level was determined by the number of cells with CA, then divided by 200, producing the CA percentage.

### Western blot analysis

Cells were lysed in radioimmunoprecipitation assay buffer (150 mM NaCl, 50 mM Tris–HCl, pH 7.2, 1% Triton X-100%, and 0.1% SDS) (Solarbio Life Sciences) containing a protease inhibitor cocktail (Sigma–Aldrich). Proteins were separated by polyacrylamide gel electrophoresis and transferred to a polyvinylidene difluoride membrane. After blocking for 1 h at room temperature with Tris-buffered saline with Tween-20 (TBST) containing 0.05% (v/v) Tween-20% and 5% (w/v) nonfat milk, the membrane was incubated with primary antibody overnight at 4 °C, followed by washes (3 × 15 min) with TBST containing 0.05% Tween-20. Subsequently, the membrane was incubated with horseradish peroxidase–conjugated secondary antibody for 1 h at room temperature, followed by washes (TBST; 3 × 15 min). ECL reagent (Thermo Biosciences) was used to visualize protein bands, which were captured by ChemiScope 6000 chemiluminescence imaging system (Clinx) and analyzed using ImageJ software.

### Wound healing and transwell assays

A wound healing assay was performed to assess the level of migration. Cells were cultured in 96-well plates to ∼90% confluence. Wounds were generated by scratching in the middle of each well and further cultured without FBS. Pictures were captured under a microscope, and the migrated distance of the cells, which indicated the level of migration, was analyzed using the ImageJ software. The Transwell assay was for the measurement of invasion. The inner well bottom was covered with rat tail collagen I (BD Bioscience). Cell suspension (5 × 10^4^ cells in 200 μl of DMEM without FBS) and 500 μl of DMEM with 10% FBS were added to the upper and lower chambers, respectively. After 15 h, the migrated cells were stained with hematoxylin and counted using the ImageJ software. Transwell without collagen was used for the measurement of migration of the cells with and without CA, for which centrosomes of the migrated cells were counted after immunofluorescent staining.

### Animal model

Approval was obtained from the Animal Ethics Committee of Jiangsu Normal University for the experimental protocol involving the use of mice. BALB/c nu/nu mice aged 6 weeks were procured from the Institute of Laboratory Animal Science and kept in a pathogen-free environment at 28 °C with 12-h light and dark cycle and fed ad libitum. The cancer cells (wildtype, empty vector, or *CEP57L1*-knockdown (1 × 10^7^ cells/ml, 100 μl) were injected into the splenic tip. Mice were assigned to different experimental groups: (1) wildtype group, treated with saline, BSA, or AGEs; (2) empty vector group, treated with BSA or AGEs; and (3) CEP57L1 knockdown group, treated with BSA or AGEs. BSA and AGEs were given i.p., both at a dose of 30 mg/kg/d for 40 days. By the end of treatment, animals were sacrificed after anesthesia, and the number of liver metastatic lesions was recorded. The expression of CEP57L1 in metastatic tumors from different groups was quantified using an immunohistochemical assay.

### Transcriptomic profiling

Total RNA was isolated using the Trizol reagent (Invitrogen Life Technologies), after which the concentration, quality, and integrity of RNA were determined using a NanoDrop spectrophotometer (Thermo Scientific). Sequencing library generation and the transcriptome data analyzation were performed according to our previous publication (37).

### RT–PCR/quantitative PCR analyses

RT–PCR was completed by reverse transcription using M-MLV reverse transcriptase (Transgene Biotech). GAPDH was used as a positive and loading control. RT–PCR products were examined on 1% agarose gels. Primer sequences used were provided in Table S4. Quantitative PCR (qPCR) was performed using ViiA 7 Real-Time PCR System (ThermoFisher) with TransStart Top Green qPCR SuperMix (Transgene Biotech). mRNA expression was normalized to the reference housekeeping gene GAPDH.

### Knockdown of protein level using siRNA

CEP57L1 and PLK4 siRNA species were purchased from GenePharma company. The sequences are presented in Table S5. For siRNA transfection, 5 × 10^4^ cells were seeded in a 6-well plate and cultured for 24 h and then transfected with 50 nM siRNA oligonucleotides using siRNA-mate transfection reagent (GenePharma), according to the manufacturer's instructions. The protein level was evaluated by Western blot analysis 36 h after transfection.

### The construction of overexpression plasmids

The entire coding regions of KLF5, KLHL13, CUL3, and CEP57L1 were obtained from HCT116 cell complementary DNA using RT–PCR. Primers for these cloning steps are listed in Table S6. The KLF5 gene was inserted into the pEGFP-C1 plasmid vector using EcoRI–KpnI sites. Whereas, KLHL13 and CUL3 were inserted into pCDNA3.1-GST and pCDNA3.1-His plasmids with EcoRI–KpnI sites, respectively. Wildtype and truncated CEP57L1 mutants (CEP57L1 1–250 N-terminal residues or 251–460 C-terminal residues) were separately subcloned into pCDNA3.1-His plasmids with EcoRI–KpnI sites. Overexpression constructs were transfected into HCT116 cells with Vigofect (Vigorous Biotechnology) following the manufacturer’s instructions. Gene overexpression was examined at two time points, 24 and 36 h after transfection, using qPCR and Western blot analyses. The CA level was obtained 48 h after transfection.

### Chromatin immunoprecipitation assay

Chromatin immunoprecipitation assay (Beyotime Biotechnology) was performed to examine the binding of KLF5 to promoters of KLHL13 and CUL3, accordingly to the manufacturer’s instructions. The amounts of immunopurified DNA were quantified using qPCR, and primer sequences are listed in Table S4.

### Dual-luciferase reporter assay

Dual-luciferase reporter vectors (pGL3-basic and phRL-TK) were purchased from Promega. Four constructs were produced.

1. -2000∼0 of KLHL13 promoter, designated as pGL3-KLHL13-promoter, which contained the KLF5 binding site(s);
2. -2000∼0 of CUL3 promoter, named as pGL3-CUL3-promoter, which contained the KLF5 binding site(s);
3. The mutated promoter sequence of KLHL13 (−2000∼-600), named as pGL3-KLHL13-promoter-mu, which was short of the binding site(s).
4. The mutated promoter sequence of CUL3 (−2000∼-100), named as pGL3-CUL3-promoter-mu, which was short of the binding site(s).

Each of the constructs was transfected into HCT116 cells with phRL-TK and pEGFP-KLF5 for quantification of the fluorescence intensity, which represented the binding level between KLF5 and promoter(s), using a dual-luciferase reporter assay system (Promega) and Synergy H4 Hybrid Re Multifunction microplate reader (BioTek) at 350 to 700 nm for 5 s, accordingly to the instructions of the manufacturers.

### Recruitment of patients

All human studies reported in this article were conducted in accordance with the principles of the Declaration of Helsinki. Approval was obtained from the Clinical Ethics Committee of Linyi People’s Hospital (Linyi, Shandong, China). A total of 212 local colorectal cancer patients were recruited consecutively in a diabetic–nondiabetic paired manner; they were matched in age (different within 3 years), sex, and cancer site and stage. Blood samples were collected and centrifuged to obtain serum (3000 rpm, 10 min). The biochemical variables were measured in a Beckman Coulter AU5821 autoanalyzer (Beckman Instrument). Approximately 1.5 μl of serum was loaded onto the machine to quantify glucose, HDL-C, LDL-C, and total cholesterol using the primary/secondary wavelengths at 340 nm and 660 nm, 600 nm and 700 nm, and 540 nm and 600 nm, respectively. In addition, 1.6 t 2 μl of serum was loaded to determine the level of triglyceride at 660/800 nm. Clinical demographics are included in Table S3. The colon adenocarcinoma data that included the clinical and genomic information of colorectal cancer patients were retrieved from TCGA (http://cancergenome.nih.gov/). Association between the overall survival and gene expression was analyzed in the TCGA Kidney Renal Clear Cell Carcinoma project. The patients were included with a cutoff set as the median; both low and high cutoffs were at 50%. The hazard ratio was calculated based on the Cox proportional hazards model.

### Immunohistochemical assay

Tissues were fixed in 10% formalin overnight. Sections were made using paraffin microtomy and baked at 65 °C overnight. Slides were deparaffinized in xylene and rehydrated sequentially in ethanol. Antigen retrieval was completed in unmasking solution, accordingly to the manufacturer's instructions. Slides were quenched in hydrogen peroxide (0.3–3%) to block endogenous peroxidase activity, washed in automation buffer (3 × 5 min), blocked in 5% FBS for 1 h at room temperature, incubated overnight at 4 °C with primary antibody diluted in blocking buffer, washed, dehydrated in ethanol, cleared with xylenes, incubated with a horseradish peroxidase–conjugated secondary antibody, and then stained with 3-3'-diaminobenzidine. Finally, the slides were mounted with Permount and captured with a brightfield microscope.

### Statistical analysis

The experiments were performed in triplicate. All data were presented in mean ± SD, unless otherwise specified. Statistical analysis was performed using the IBM SPSS Statistics (27.0) computer software package. Student’s *t* test was used for the comparison between two groups. Multigroup comparisons were performed by one-way ANOVA. A *p* < 0.05 was considered to be statistically significant. ∗*p* < 0.05, ∗∗*p* < 0.01, and ∗∗∗*p* < 0.001, compared with the control group(s). #*p* < 0.05 and ##*p* < 0.01, compared with the treated group. The method of Pearson's correlation analysis was employed for the correlation between fasting blood glucose concentration and CA as well as that between the expression levels of KLF5 and KLHL13, CUL3, or CEP57L1.

## Ethical statement

The clinical sample collection approval was obtained from the Clinical Ethics Committee of Linyi People’s Hospital (Linyi, Shandong, China; no.: YX200658). The animal experimental approval was obtained from the Animal Ethics Committee of Jiangsu Normal University (no.: JSNU-IACUC-2024038).

## Data availability

The datasets presented in this study can be found in online repositories. The names of the repository/repositories and accession number(s) can be found below: https://figshare.com/s/20956a8398c1a821a428.

## Supporting information

This article contains supporting information.

## Conflict of interest

The authors declare that they have no conflicts of interest with the contents of this article.
