# Supplementary material for: AGEs promote the metastasis of colorectal cancer cells via centrosome amplification by KLF5–CEP57L1 axis
Source: J Biol Chem. 2025 Dec 22;302(2):111098. doi: 10.1016/j.jbc.2025.111098 (PMC12828400; doi:10.1016/j.jbc.2025.111098)
Supplement: Supplementary Material 1 [file mmc1.docx]

**Supplementary Tables**

| Table 1. The predicated binding sites of KLF5 on the promoter sequence of KLHL13 | | | | | |
| --- | --- | --- | --- | --- | --- |
| Number | Score | Start | End | strand | Predicted sequence |
| 1 | 12.518292 | 1427 | 1436 | - | CCCCCTCCCT |
| 2 | 12.518292 | 1938 | 1947 | + | CCCCCTCCCT |
| 3 | 12.518292 | 1966 | 1975 | + | CCCCCTCCCT |
| 4 | 11.641731 | 1986 | 1995 | + | CCCACACCCT |
| 5 | 11.149876 | 1959 | 1968 | + | TTCCCGCCCC |
| 6 | 10.186536 | 1942 | 1951 | + | CTCCCTCCCC |
| 7 | 9.693543 | 1433 | 1442 | - | ATCCCTCCCC |
| 8 | 6.445924 | 1924 | 1933 | + | TCCCCCCCCC |
| 9 | 6.202313 | 1562 | 1571 | + | GCCCCGCCGT |
| 10 | 5.9855843 | 1909 | 1918 | + | CCCCCGCCCG |

| Table 2. The predicated binding sites of KLF5 on the promoter sequence of CUL3 | | | | | |
| --- | --- | --- | --- | --- | --- |
| Number | Score | Start | End | strand | Predicted sequence |
| 1 | 13.540034 | 1541 | 1550 | - | CCCCCACCCC |
| 2 | 13.378423 | 1956 | 1965 | - | TCCCCACCCA |
| 3 | 11.082259 | 1018 | 1027 | - | ACCACACCCA |
| 4 | 5.9904165 | 1085 | 1094 | - | CCTCCGCCTC |
| 5 | 5.2157702 | 824 | 833 | - | TCTCCTCCTA |
| 6 | 4.7109747 | 1841 | 1850 | - | GCAACTCCCC |
| 7 | 4.642753 | 1539 | 1548 | - | CCCACCCCCC |
| 8 | 4.5090914 | 1745 | 1754 | - | TCCCCAGCCA |
| 9 | 3.1227067 | 1723 | 1732 | + | TCCACAGCCT |
| 10 | 3.0562272 | 224 | 233 | + | TCCAAACCCA |

| Table 3. The clinical characteristic of intestinal cancer patients with or without diabetes. | | | | |
| --- | --- | --- | --- | --- |
| Patients | Total (n=212) | Non-diabetes (n=106) | Diabetes (n=106) | p-value |
| Age | 64.76 (63.05-66.47) | 62.08 (59.71-64.46) | 67.46 (65.07-69.84) | 0.0018 |
| Sex | M:F=1:1.08 | M:F=1:1.12 | M:F=1.04:1 | ns |
| Fasting blood glucose - FBG | 7.00 (6.61-7.38) | 5.30 (4.96-5.65) | 8.64 (8.13-9.15) | 2.2064e-21 |
| Systolic blood pressure - SBP | 130.50 (128.25-132.76) | 125.83 (123.36-128.30) | 135.18 (131.57-138.79) | 3.3105e-05 |
| Diastolic blood pressure - DBP | 76.70 (75.36-78.03) | 75.03 (73.27-76.78) | 78.38 (76.38-80.38) | 0.0131 |
| Total cholesterol - TC | 3.97 (3.83-4.11) | 3.88 (3.71-4.05) | 4.06 (3.84-4.29) | 0.1932 |
| Triglycerides - TG | 1.32 (1.24-1.41) | 1.27 (1.16-1.37) | 1.37 (1.24-1.51) | 0.2067 |
| High-density lipoprotein (HDL) | 0.99 (0.95-1.03) | 0.98 (0.93-1.03) | 1.00 (0.93-1.07) | 0.6392 |
| Low-density lipoprotein (LDL) | 2.48 (2.38-2.59) | 2.52 (2.40-2.65) | 2.45 (2.27-2.62) | 0.4898 |
| Data is expressed in mean (95% confidence interval)  p-value, compared between diabetic and non-diabetic patients. | | | | |

| Table 4. List of primers used for quantification of specific gene expression | | |
| --- | --- | --- |
| *Gene* | *Forward (5’-3’)* | *Reverse (5’-3’)* |
| KLF5 | CCTGGTCCAGACAAGATGTGA | GAACTGGTCTACGACTGAGGC |
| KLHL13 | CTCCCTTTTGAGCGTCTTGCCTTCG | GTGGGGCTCACTCATTTTGGCAACA |
| CUL3 | TTGGAGGTGCACAAGTAACTGGCTC | TGTTGGTTTACCACAGGCGAGGGAC |
| CEP57L1 | AGCTCAGCCCAGTCTCGTTGTACTC | GGATATGTGGGTCACGATGTTGCCT |
| PLK4 | GCTTCTCCAACACAGGCACCAATCC | GAAGACACTCCTGCCTGCACAACCA |
| CEP20 | ATGGCGACTGTGGCAGAGTTGAAGG | CCTCCTTTCTTAGGTGGTCATCTGC |
| Aurora B | CAGAAGAGCTGCACATTTGACG | CCTTGAGCCCTAAGAGCAGATTT |
| GAPDH | CACCATCTTCCAGGAGCGAGATCCC | CCATCACGCCACAGTTTCCCGGAGG |
| KLHL13 ChIP (-1 to -100; including the Site 2, 3,4, 5, 6, 8, and 10) | CCATGTTTCCCCCGCCCGCGC | TCACCAGGGTGTGGGGGGGCTGCT |
| KLHL13 ChIP (+50 to -50; including the Site 3, 4, and 5) | CAACTGGGTTCCCGCCCCCCTC | GCCCTGATCAGACTCTTTATTGCAG |
| KLHL13 ChIP (-50 to -200; including the Site 2, 6, 8, and 10) | CTACAGCCTGGGAGTTCCGTTCGGA | GGGAGGGAGGGGGCAGTGG |
| KLHL13 ChIP (-65 to -200; including the Site 8 and 10) | CTACAGCCTGGGAGTTCCGTTCGGA | GTGGGGGGGGGAGCGCGCGG |
| KLHL13 ChIP (-400 to -600; including the Site 1, 7 and 9) | TCTTTCTTAATGTTGTGTACTGCAGCAG | CCAGCCGTGGCCTCGGCCTCCTC |
| CUL3 ChIP (-450 to -600; including the Site 1 and 7) | ACTATGTCTGTGATAATTTGTTATA | ACACCCCCACCCCCCAAAACTT |
| CUL3 ChIP (-470 to -600; including non Site) | ACTATGTCTGTGATAATTTGTTATA | TTTTGTTATTGTTTATTATTTCCTTAATTTTG |
| CUL3 ChIP (-1 to -200; including the Site 2 and 6) | GCACATCTTACGTGGCAGGAGCAG | GATGTAGAACCCAAATGATATAGTT |
| CUL3 ChIP (-900 to -1000; including the Site 3 and 4) | ACACCTGTAATCCCAGCACTTTGG | CCTCAACCTCCGCCTCCTGGGTTCA |
| CUL3 ChIP (-1100 to -1200; including the Site 5) | GCCTAATGTAATCACAGGGTTCTTA | AAGCCACTGCGCCTGGCCTCCTCTTG |
| CUL3 ChIP (-200 to -300; including the Site 8 and 9) | AACTGTTTAATTGGTTCACAGTTCC | CTGCTTCCCCTTCACCTTCTGCCAT |
| CUL3 ChIP (-1700 to -1800; including the Site 1 and 7) | ATTCTTCAAGTTACTTTTAATATTCC | ACAGTATAATTTTATATCTTAATTCT |

| Table 5. List of siRNA or shRNA sequences used in this study. | |
| --- | --- |
| *siRNA* | *Oligomers (5’-3’)* |
| CEP57L1 | GCUUUCAUCCAAUACGAGUTT |
| Vimentin | GCAAGUAUCCAACCAACUUTT |
| CEP57L1 shRNA | Top stand: CACCGGAGAGAACACAAGCTGAAGACGAATCTTCAGCTTGTGTTCTCTCC  Bottom stand: AAAAGGAGAGAACACAAGCTGAAGATTCGTCTTCAGCTTGTGTTCTCTCC |

| Table 6. List of primers used for overexpression vector construction. | | | |
| --- | --- | --- | --- |
| *Plasmid name* | *Gene* | *Forward (5’-3’)*  *Reverse (5’-3’)* | *Restriction enzyme* |
| pEGFP-C1 | KLF5 | CGGAATTCATGGAGAAGTATCTGACACCTCAGC  TTAAGGTACCTCAGTTCTGGTGCCTCTTCATATGCAG | *Eco*R Ⅰ  *Kpn* Ⅰ |
| pCDNA3.1-GST | KLHL13 | GCAAGCTTATGATGAGAGTTCAAACCTTAAGAG  TGGAATTCTTAAGGTGCAGAAAGAGGGGACTCT | *Hind* Ⅲ  *Eco*R Ⅰ |
| pCDNA3.1-his | CUL3 | GCAAGCTTATGTCGAATCTGAGCAAAGGCACGG  TGGAATTCTTATGCTACATATGTGTATACTTTG | *Hind* Ⅲ  *Eco*R Ⅰ |
| pCDNA3.1-his | CEP57L1 (1-250 residues) | GCAAGCTT ATGGATTCTGAATTAATGCATAG  TGGAATTCAGTTTTCTTTGAAGATTTCTTC | *Hind* Ⅲ  *Eco*R Ⅰ |
| pCDNA3.1-his | CEP57L1 (251-460 residues) | GCAAGCTTATGAAATGTATAAAGAGACGACCACC  TGGAATTCCTGTTCCCACATGATATCATCT | *Hind* Ⅲ  *Eco*R Ⅰ |

**Supplementary Figures**

**
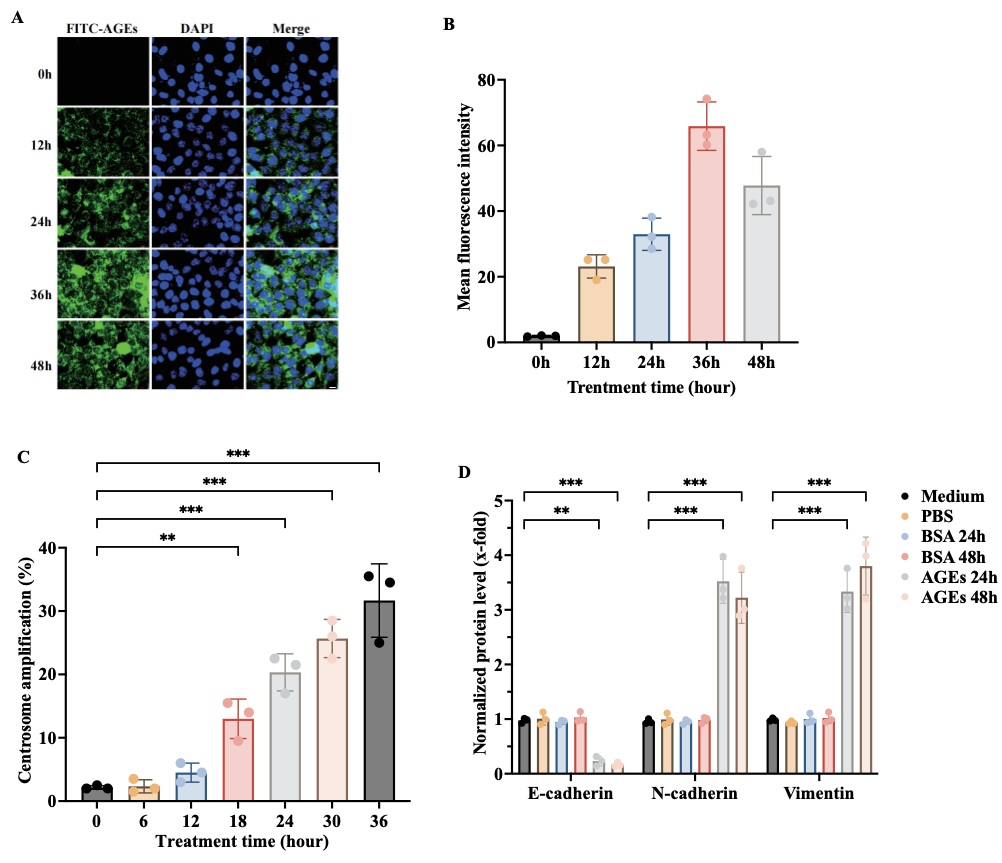
**

**Fig. 1.​​ AGEs enter colorectal cancer cells and induce centrosome amplification (CA) and epithelial–mesenchymal transition (EMT).** (A)​​ Representative immunofluorescence images of HCT116 cells treated with FITC-conjugated AGEs (green) showing cellular uptake. Nuclei were stained with DAPI (blue). Scale bar: 20 μm. (B)​​ Quantification of FITC-AGE fluorescence intensity from (A), confirming excellent cellular uptake (visualized by FITC conjugation) and stability (<10% degradation at 48h vs. 36h; n = 3 independent experiments). (C)​​ Time-dependent induction of CA in HCT116 cells treated with AGEs (300 μg/ml). CA frequency was assessed by γ-tubulin staining (n ≥ 200 cells per condition). (D)​​ Quantification of Western blot signals from Fig. 1G showing expression levels of EMT markers (E-cadherin, N-cadherin, vimentin) normalized to GAPDH. **: p<0.01 and ***: p<0.001, compared to the medium or BSA groups.

**
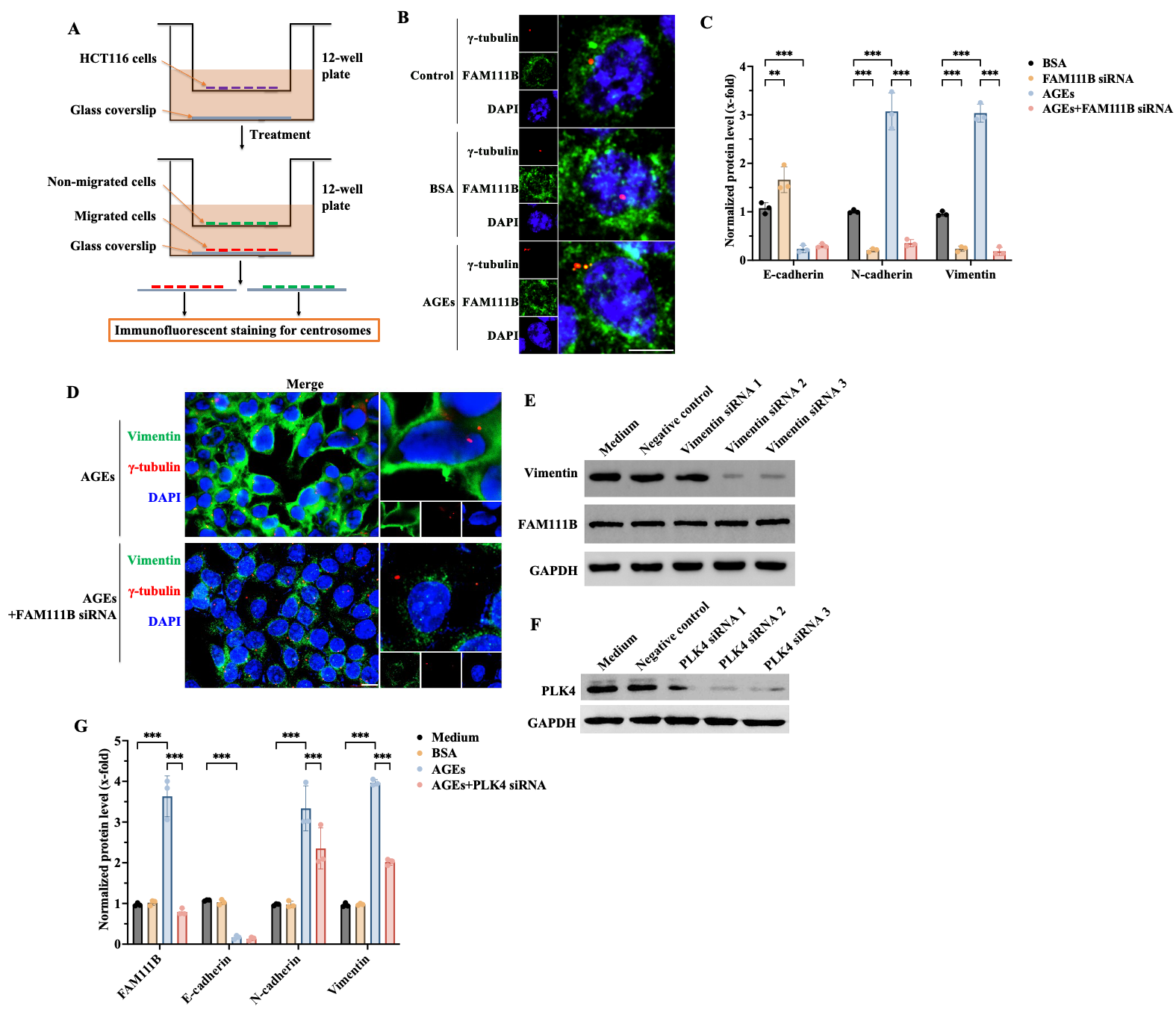
**

**Figure 2.** AGEs promote migration and invasion through a CA-FAM111B-EMT pathway. (A)​​ Schematic diagram of the Transwell-based method used to collect migrated and non-migrated HCT116 cells for CA analysis. (B)​​ Representative immunofluorescence images showing enhanced centrosomal localization of FAM111B (green) upon AGEs treatment; centrosomes were stained with γ-tubulin (red) and nuclei with DAPI (blue). Scale bar: 20 μm. (C)​​ Quantification of EMT marker expression (E-cadherin, N-cadherin, vimentin) from Western blots in Fig. 2H, normalized to GAPDH. (D)​​ FAM111B knockdown attenuates AGE-induced vimentin accumulation at the sub-membranous region. (E)​​ Vimentin knockdown using siRNA does not alter FAM111B protein levels. (F)​​ PLK4 knockdown using siRNA. (G)​​ Quantification of Western blot signals from Fig. 2N. **: p<0.01 and ***: p<0.001, compared to the medium or BSA groups.


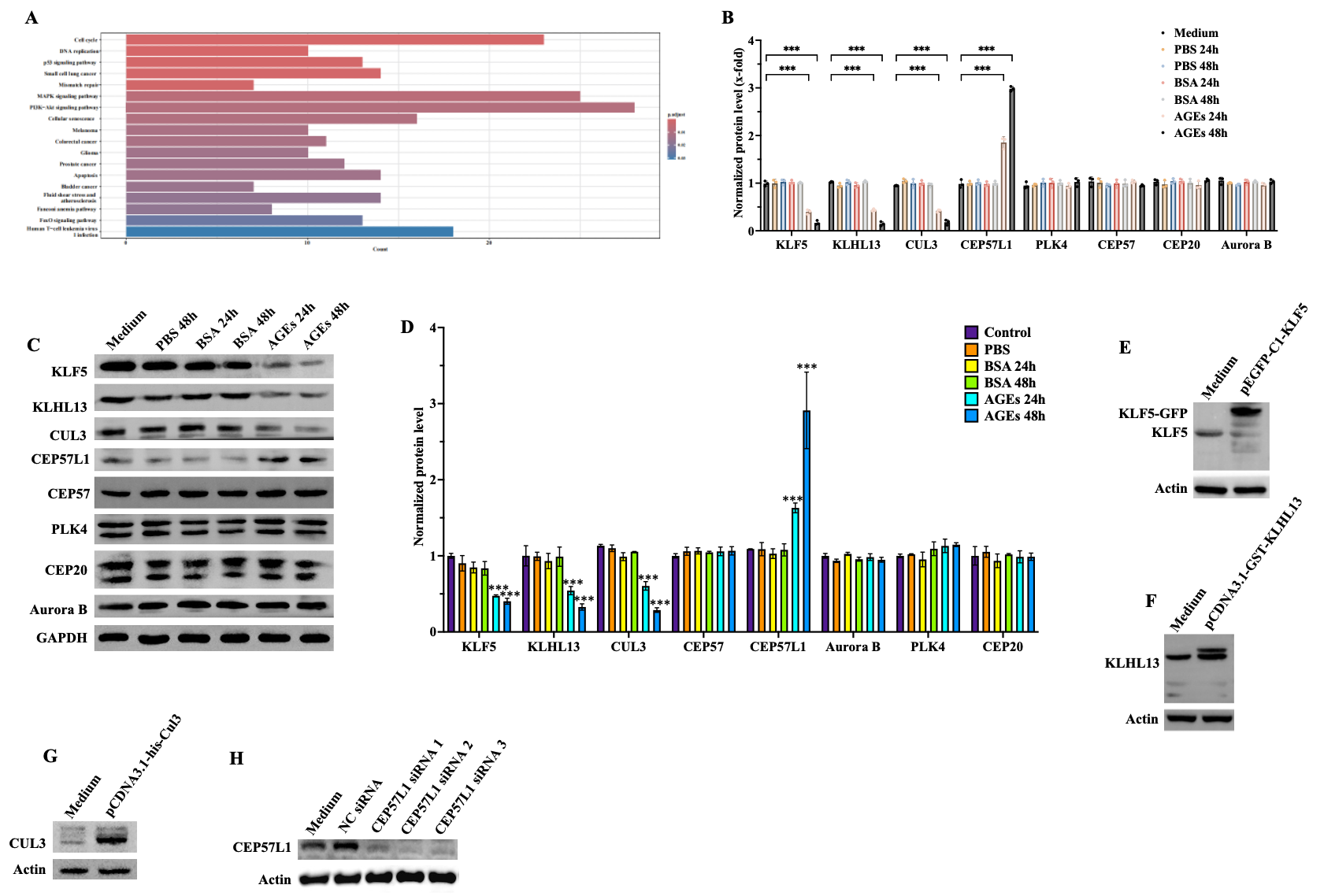


**Figure 3.** Transcriptomic profiling identifies KLF5‑KLHL13‑CUL3‑CEP57L1 as a signalling axis associated with AGE‑induced centrosome amplification. (A)​​ KEGG pathway enrichment analysis of differentially expressed genes in AGE‑treated HCT116 cells, showing "Cell cycle" and "DNA replication" as the most significantly enriched terms. (B)​​ Quantification of Western blot signals from Fig. 3E, representing protein levels of KLF5, KLHL13, CUL3, CEP57L1, CEP57, PLK4, CEP20 and Aurora B in HCT116 cells after AGE treatment. (C)​​ Western blot analysis confirming that AGEs downregulate KLF5, KLHL13 and CUL3, and upregulate CEP57L1, but do not affect CEP57, PLK4, CEP20 or Aurora B in SW620 cells. (D)​​ Quantification of Western blot signals from Fig. S3C. (E–G)​​ Overexpression of KLF5 (E), KLHL13 (F) and CUL3 (G) inhibits AGE‑induced centrosome amplification. (H)​​ Knockdown of CEP57L1 using siRNA attenuates centrosome amplification. ***: p<0.001, compared to the medium or BSA groups.


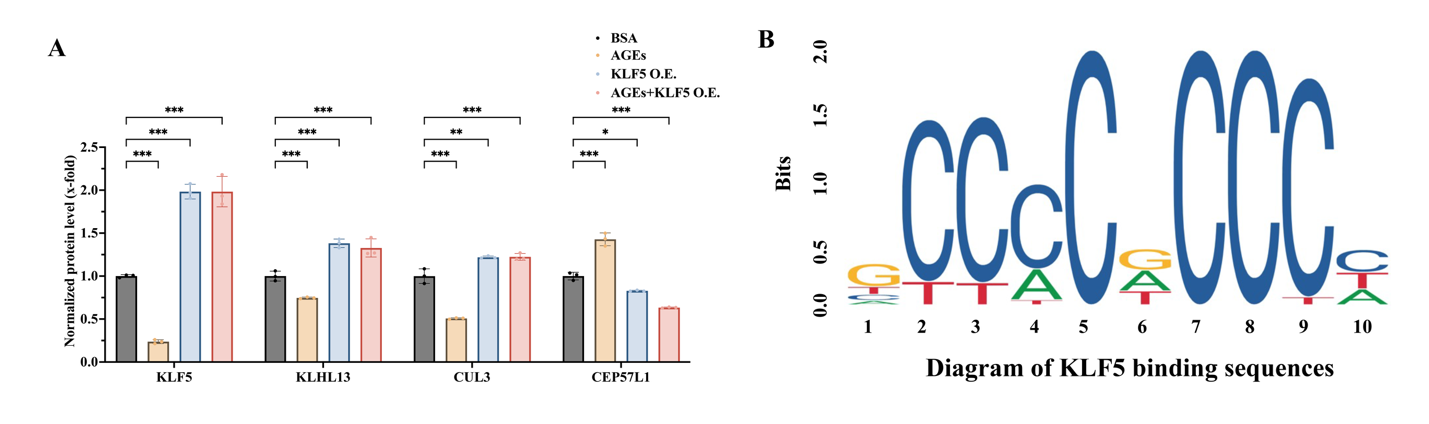


**Figure 4.** KLF5 transcriptionally regulates KLHL13 and CUL3 under AGE treatment. (**A**) Quantification of Western blot signals from Fig. 4B showing protein levels of KLF5, KLHL13, CUL3, and CEP57L1 in HCT116 cells with or without KLF5 overexpression and AGE treatment. (**B**) The diagram of KLF5 binding sequences. *: p<0.05; **: p<0.01; ***: p<0.001, compared to the medium or BSA groups.


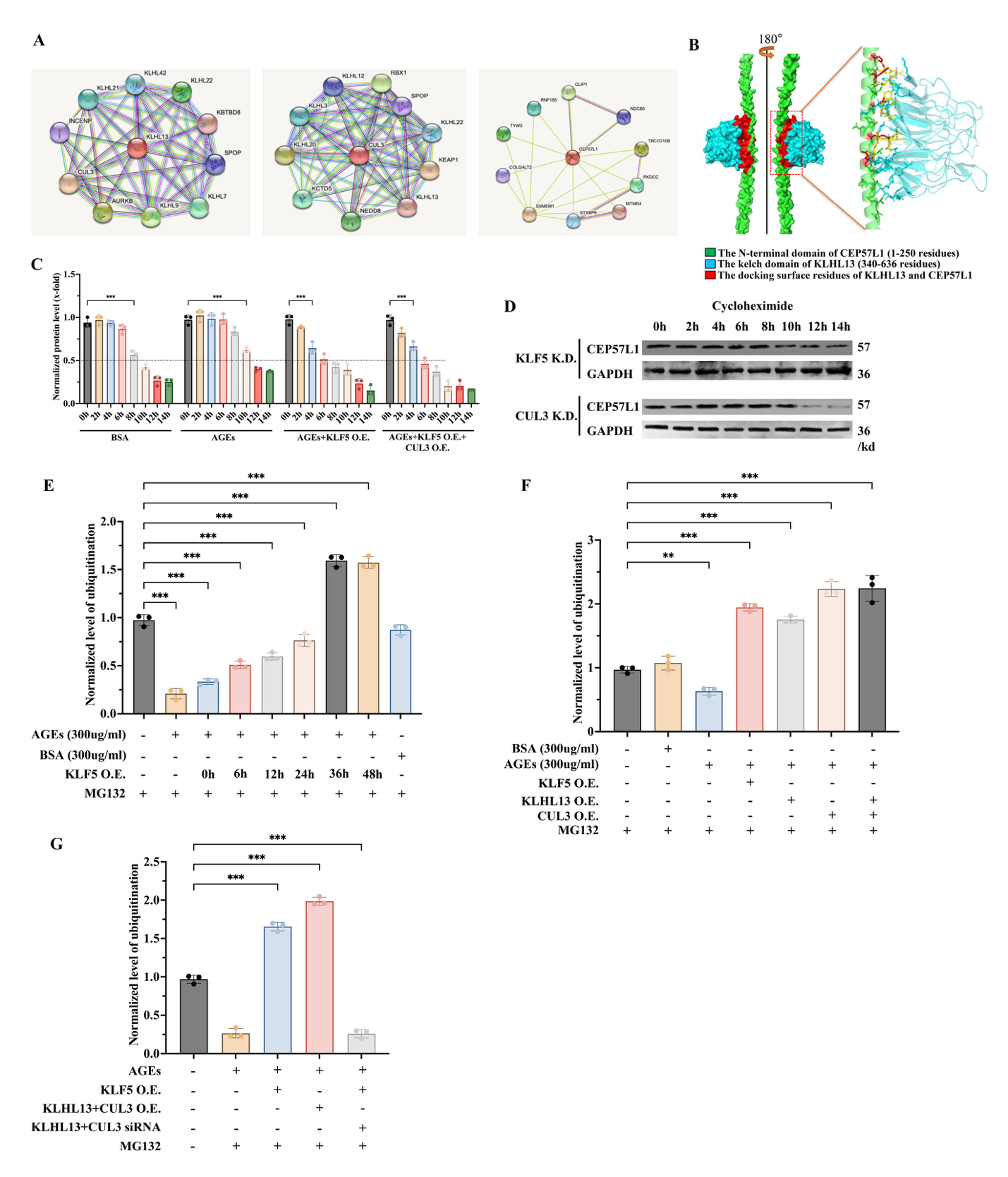


**Figure 5.** KLHL13 and CUL3 form an E3 ubiquitin ligase complex that targets CEP57L1 for degradation. (**A**) List of predicted interacting proteins for KLHL13, CUL3, and CEP57L1 from the STING database. (**B**) Predication that CEP57L1 interacted with the kelch domain of KLHL13 (340-636 residues) via its N-terminal domain including the 1-250 residues. (C) Quantification of CEP57L1 protein half-life in HCT116 cells treated with AGEs, with or without overexpression of KLF5 or KLF5+CUL3. Data were derived from cycloheximide chase assays (Fig. 5C) and normalized to GAPDH. (D) CEP57L1 half-life analysis under KLF5 or CUL3 knockdown conditions. (E-G) Quantification of CEP57L1 ubiquitination levels from Western blots in Figs. 5F–H, respectively. **: p<0.01; ***: p<0.001, compared to the medium or BSA groups.


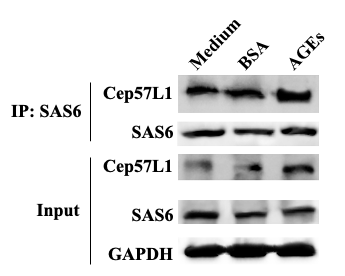


**Figure 6.** AGEs promote the interaction between CEP57L1 and the cartwheel protein SAS6. Co‑immunoprecipitation assays using an anti‑SAS6 antibody in HCT116 cells demonstrate enhanced binding between endogenous SAS6 and CEP57L1 following AGEs treatment, suggesting their cooperative role in procentriole assembly.


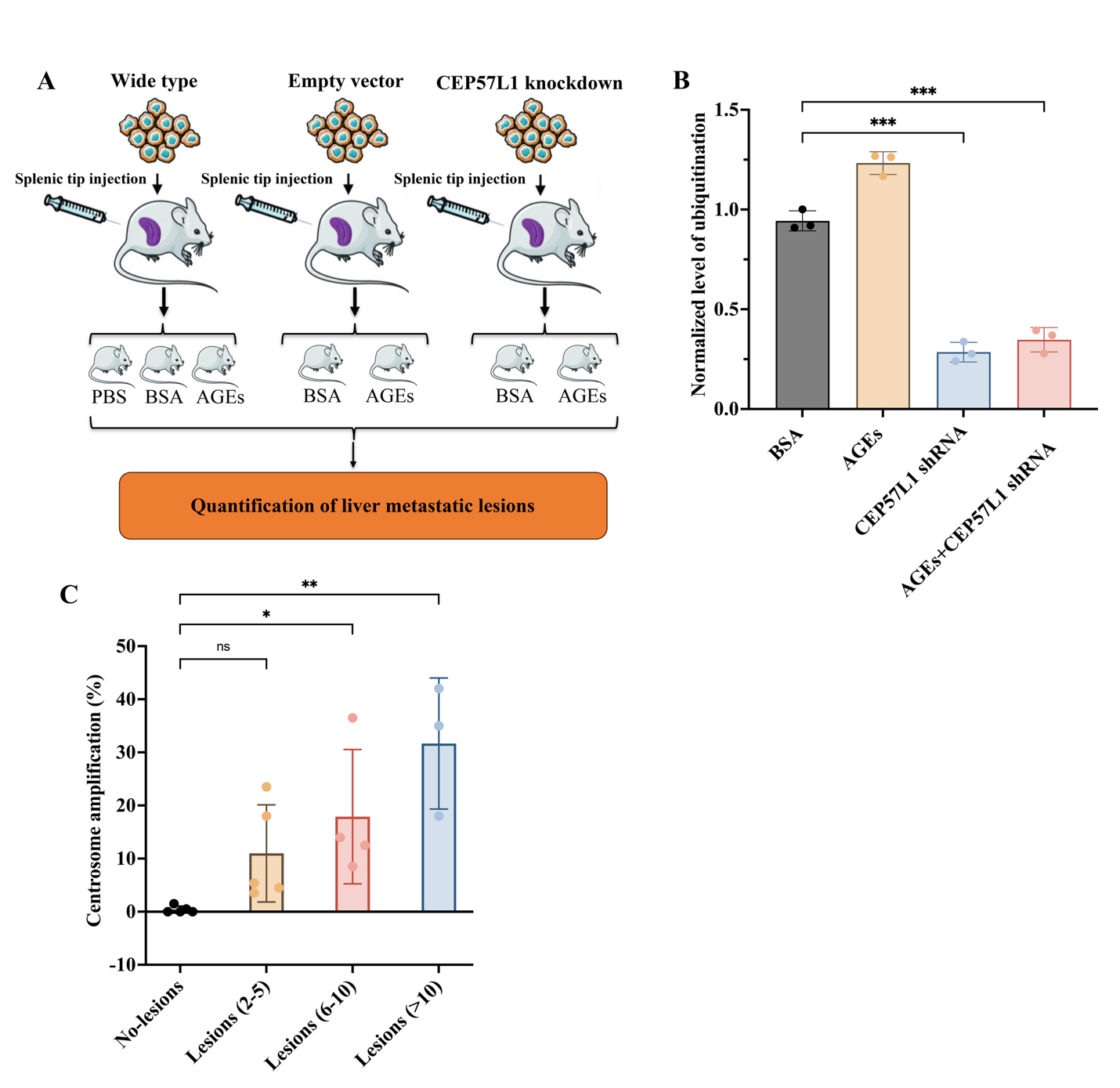


**Figure 7.** AGEs promote liver metastasis of colorectal cancer cells in a CEP57L1‑ and CA‑dependent manner *in vivo*. (A) Schematic of the experimental design: wild‑type, empty vector‑transfected, or CEP57L1‑knockdown HCT116 cells were injected into the spleen of nude mice, followed by treatment with BSA or AGEs; liver metastasis was assessed. (B) Quantification of CEP57L1 ubiquitination levels from Western blots in Figs. 7C. (C) CA frequency positively correlates with the extent of liver metastasis: livers with no metastatic nodules showed <1% CA; those with 2–5, 6–10, or >10 nodules displayed ~10%, ~18%, and ~30% of CA, respectively. ns: no significant; *: p<0.05; **: p<0.01; ***: p<0.001, compared to the medium, BSA groups or No-lesions group.

**
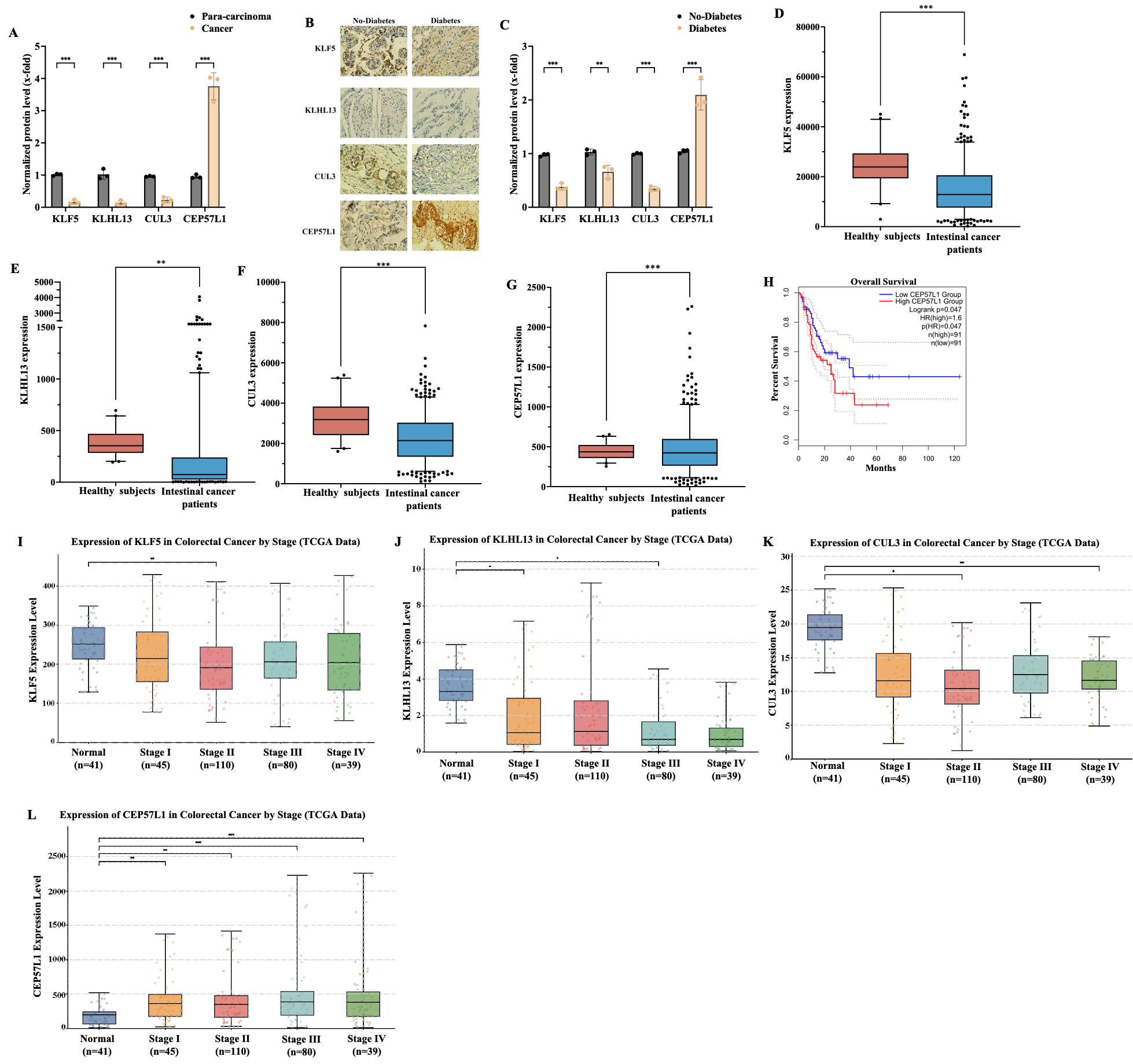
**

**Figure 8 Clinical relevance of the KLF5‑KLHL13‑CUL3‑CEP57L1 axis in colorectal cancer patients. (A)** Quantification of KLF5, KLHL13, CUL3, and CEP57L1 levels from Fig. 8F in paired cancer and adjacent non‑cancer tissues. (B) Immunohistochemical analysis revealed that KLF5, KLHL13, and CUL3 expression was lower, while CEP57L1 was higher, in cancer tissues from diabetic patients compared to non‑diabetic patients. (C) Quantification of protein expression levels from immunohistochemical staining in Fig. S8B. (D-G) The transcription of KLF5 (A), KLHL13 (B), CUL3 (C) and CEP57L1(D) were quantified in healthy subjects and intestinal cancer patients. (H) Kaplan–Meier survival analysis showed that CEP57L1 expression level was not significantly associated with overall survival in colorectal cancer patients. (I-L) The correlation between the expression levels of KLF5, KLHL13, CUL3, and CEP57L1 and CRC tumor stages was analyzed using the TCGA COAD dataset (n=315). ns: no significant; *: p<0.05; **: p<0.01; ***: p<0.001.
