# Supplementary material for: AGEs promote the metastasis of colorectal cancer cells via centrosome amplification by KLF5–CEP57L1 axis
Source: J Biol Chem. 2025 Dec 22;302(2):111098. doi: 10.1016/j.jbc.2025.111098 (PMC12828400; doi:10.1016/j.jbc.2025.111098)
Supplement: Supplementary Material 2 [file mmc2.pdf]

Figure 1G

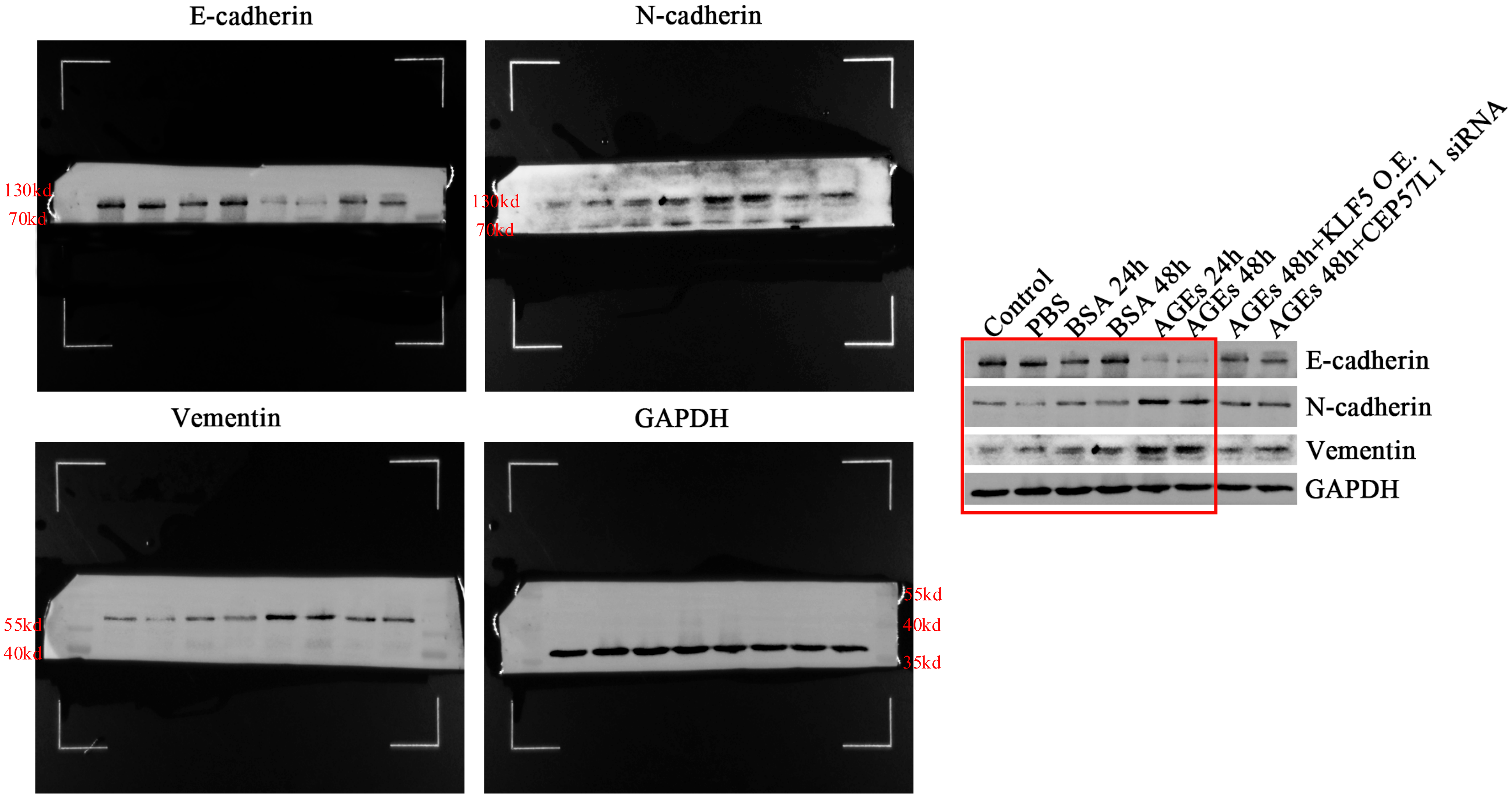

E-cadherin
N-cadherin
Vementin
GAPDH
130kd
70kd
130kd
70kd
55kd
40kd
55kd
40kd
35kd
Control
PBS
BSA 24h
BSA 48h
AGEs 24h
AGEs 48h
AGEs 48h+KLF5 O.E.
AGEs 48h+CEP57L1 siRNA
E-cadherin
N-cadherin
Vementin
GAPDH

Figure 2G

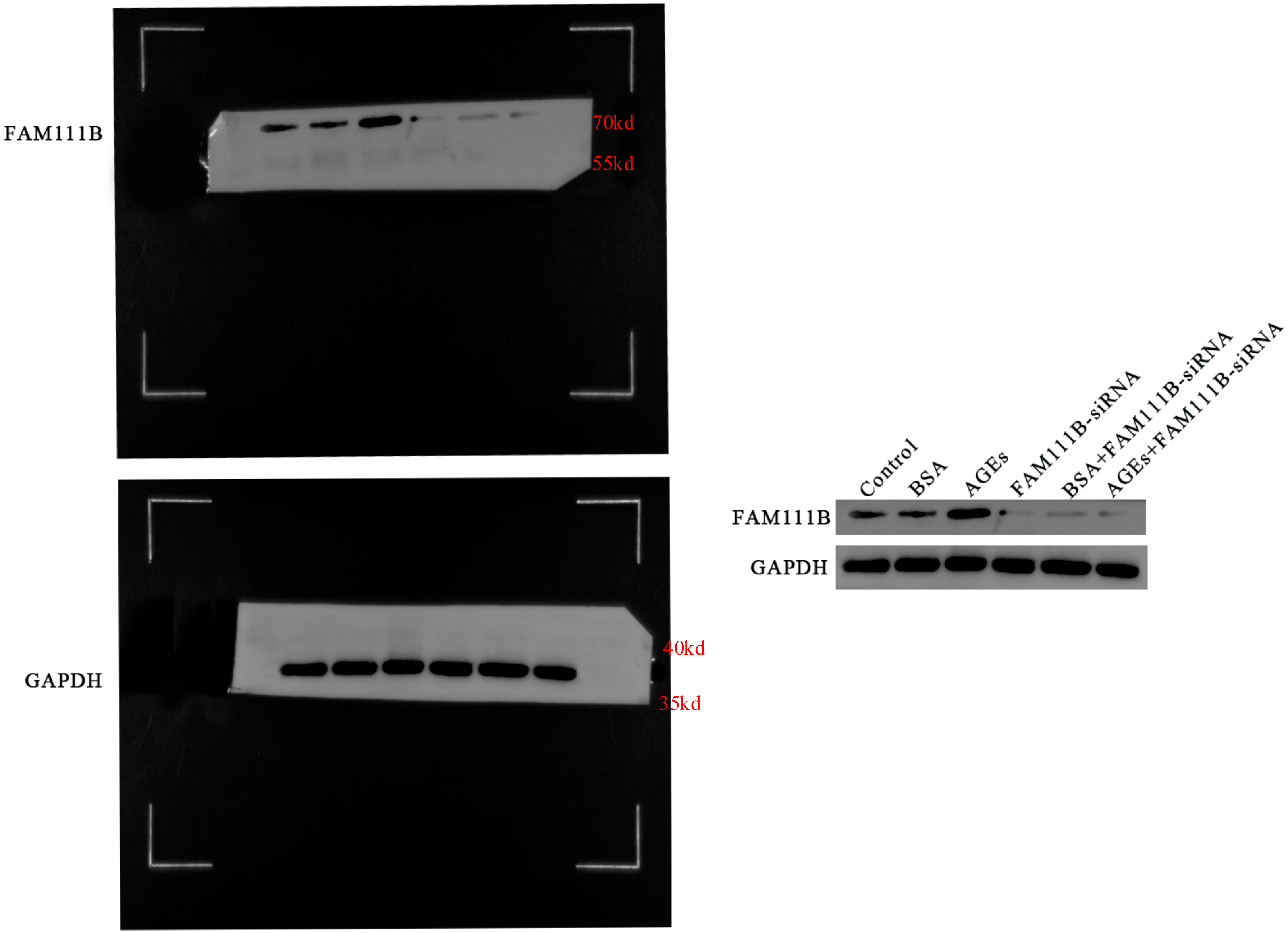
FAM111B
70kd
55kd
GAPDH
40kd
35kd
Control
BSA
AGEs
FAM111B-siRNA
BSA+FAM111B-siRNA
AGEs+FAM111B-siRNA
FAM111B
GAPDH

Figure 2H

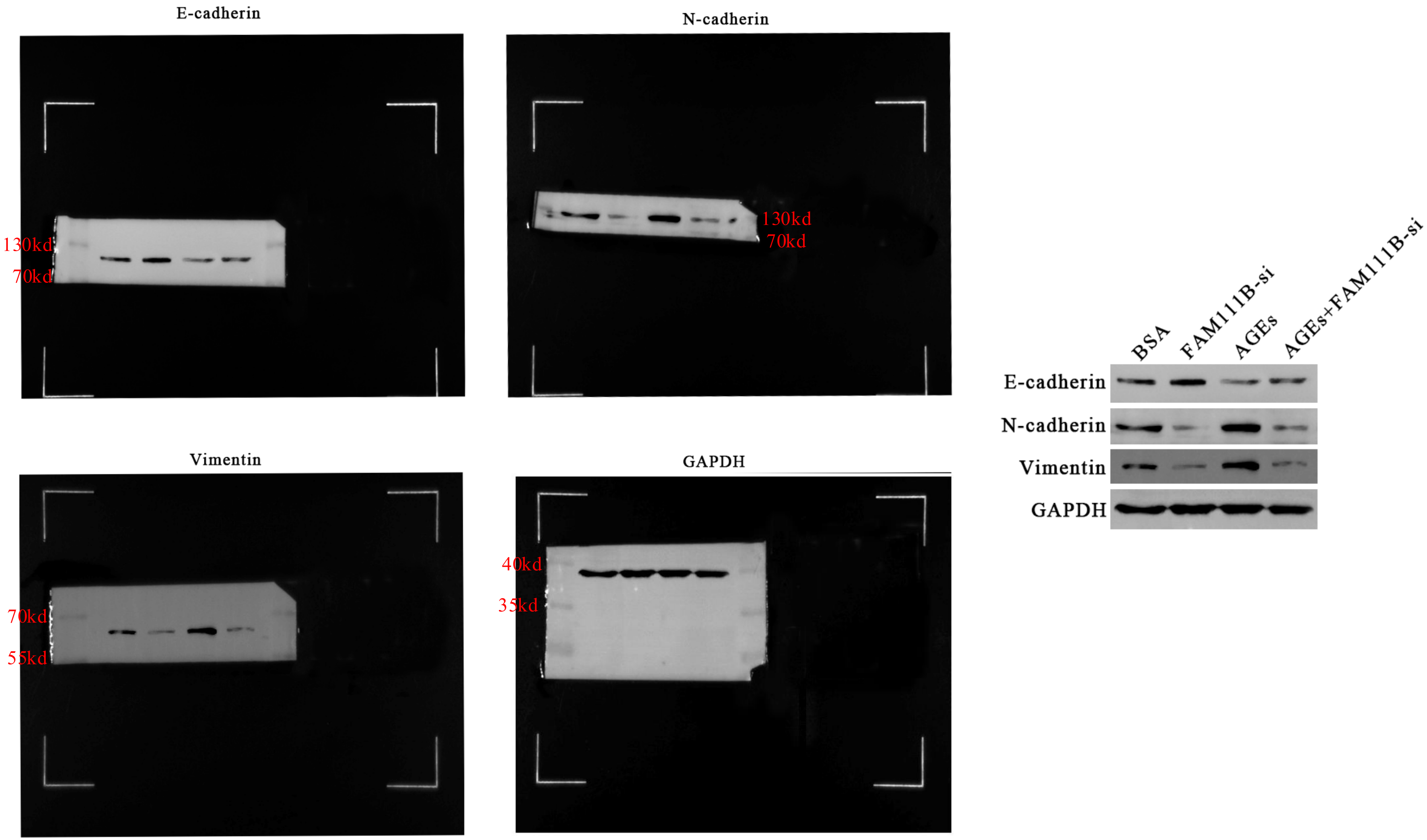
E-cadherin
130kd
70kd
N-cadherin
130kd
70kd
Vimentin
70kd
55kd
GAPDH
40kd
35kd
BSA
FAM111B-si
AGEs
AGEs+FAM111B-si
E-cadherin
N-cadherin
Vimentin
GAPDH

Figure 2M

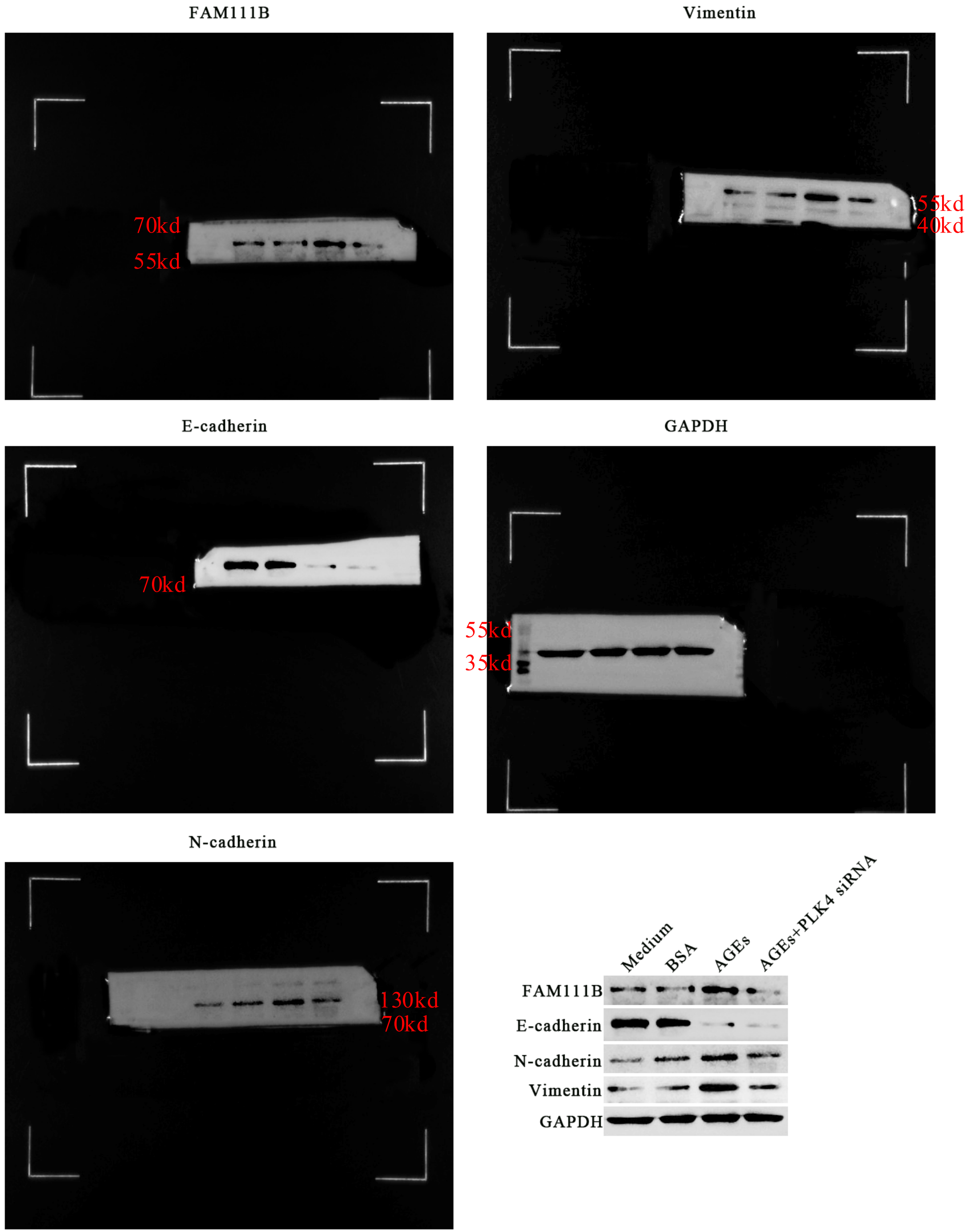
FAM111B
70kd
55kd
Vimentin
55kd
40kd
E-cadherin
70kd
GAPDH
55kd
35kd
N-cadherin
130kd
70kd
Medium
BSA
AGEs
AGEs+PLK4 siRNA
FAM111B
E-cadherin
N-cadherin
Vimentin
GAPDH

Figure 3E

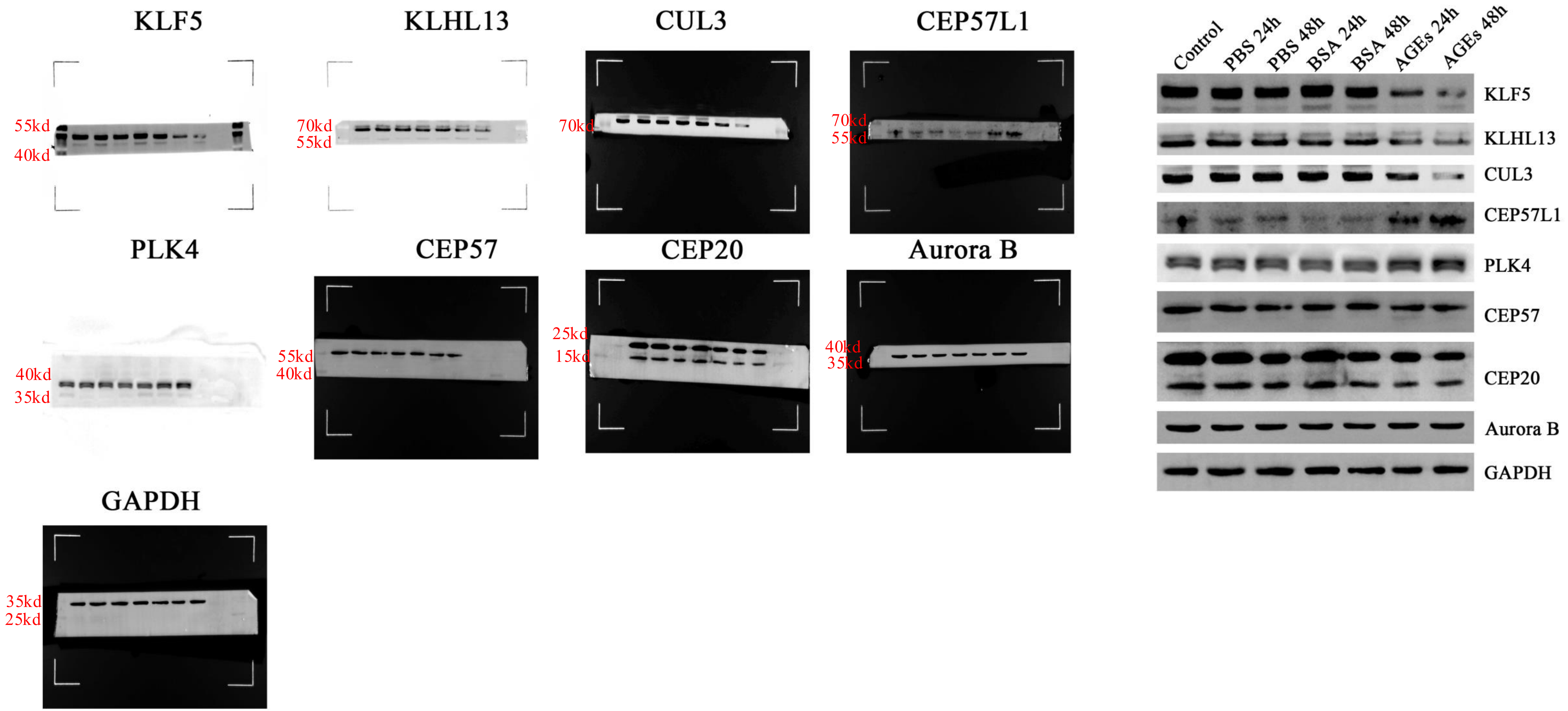
KLF5
55kd
40kd
KLHL13
70kd
55kd
CUL3
70kd
CEP57L1
70kd
55kd
PLK4
40kd
35kd
CEP57
55kd
40kd
CEP20
25kd
15kd
Aurora B
40kd
35kd
GAPDH
35kd
25kd
Control
PBS 24h
PBS 48h
BSA 24h
BSA 48h
AGEs 24h
AGEs 48h
KLF5
KLHL13
CUL3
CEP57L1
PLK4
CEP57
CEP20
Aurora B
GAPDH

Figure 4B

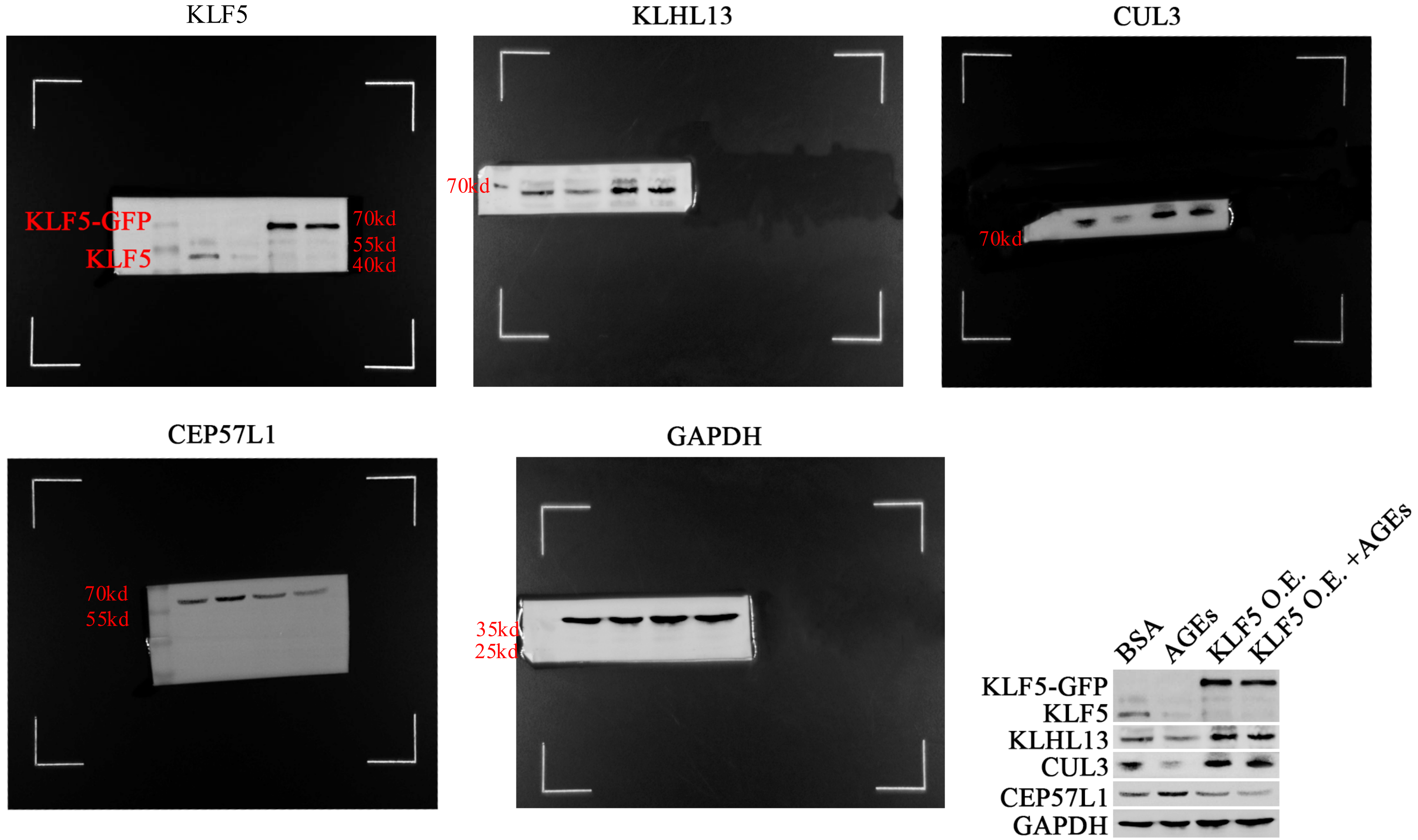
KLF5
KLHL13
CUL3
KLF5-GFP
KLF5
70kd
55kd
40kd
70kd
70kd
CEP57L1
GAPDH
70kd
55kd
35kd
25kd
BSA
AGEs
KLF5 O.E.
KLF5 O.E. +AGEs
KLF5-GFP
KLF5
KLHL13
CUL3
CEP57L1
GAPDH

Figure 5A

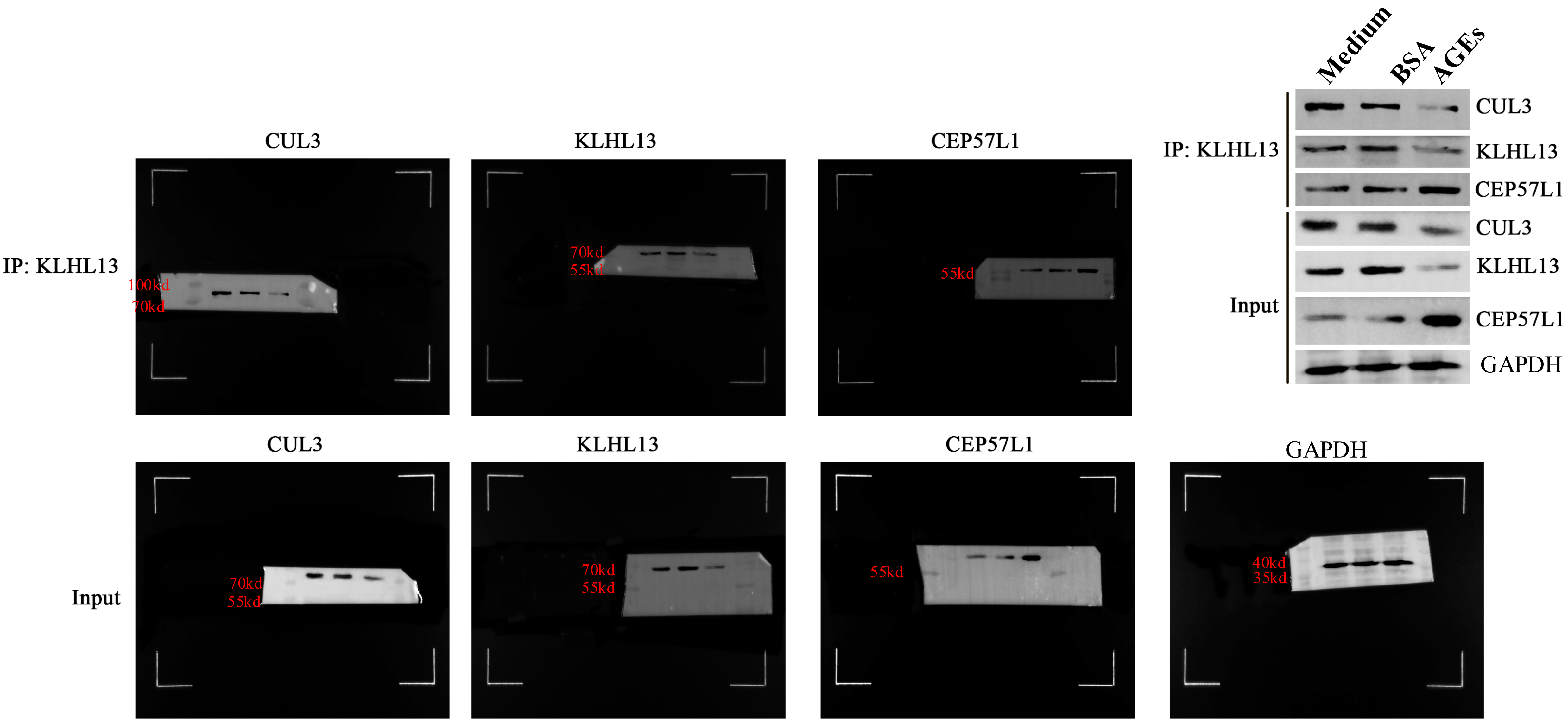

IP: KLHL13
Input
CUL3
KLHL13
CEP57L1
GAPDH
100kd
70kd
55kd
40kd
35kd
Medium
BSA
AGEs

Figure 5B

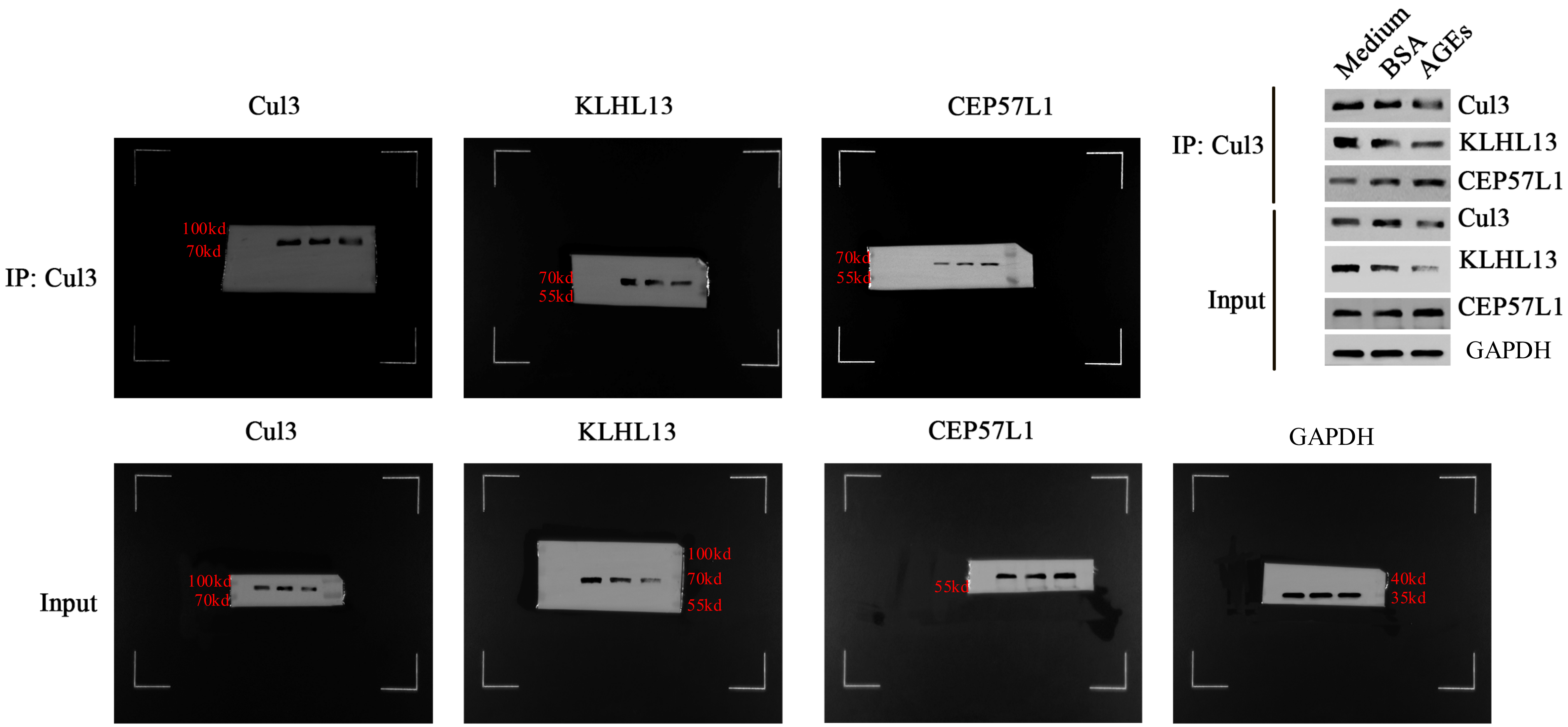

Medium
BSA
AGEs
IP: Cul3
Cul3
KLHL13
CEP57L1
Input
Cul3
KLHL13
CEP57L1
GAPDH
IP: Cul3
Cul3
100kd
70kd
KLHL13
70kd
55kd
CEP57L1
70kd
55kd
Input
Cul3
100kd
70kd
KLHL13
100kd
70kd
55kd
CEP57L1
55kd
GAPDH
40kd
35kd

Figure 5C

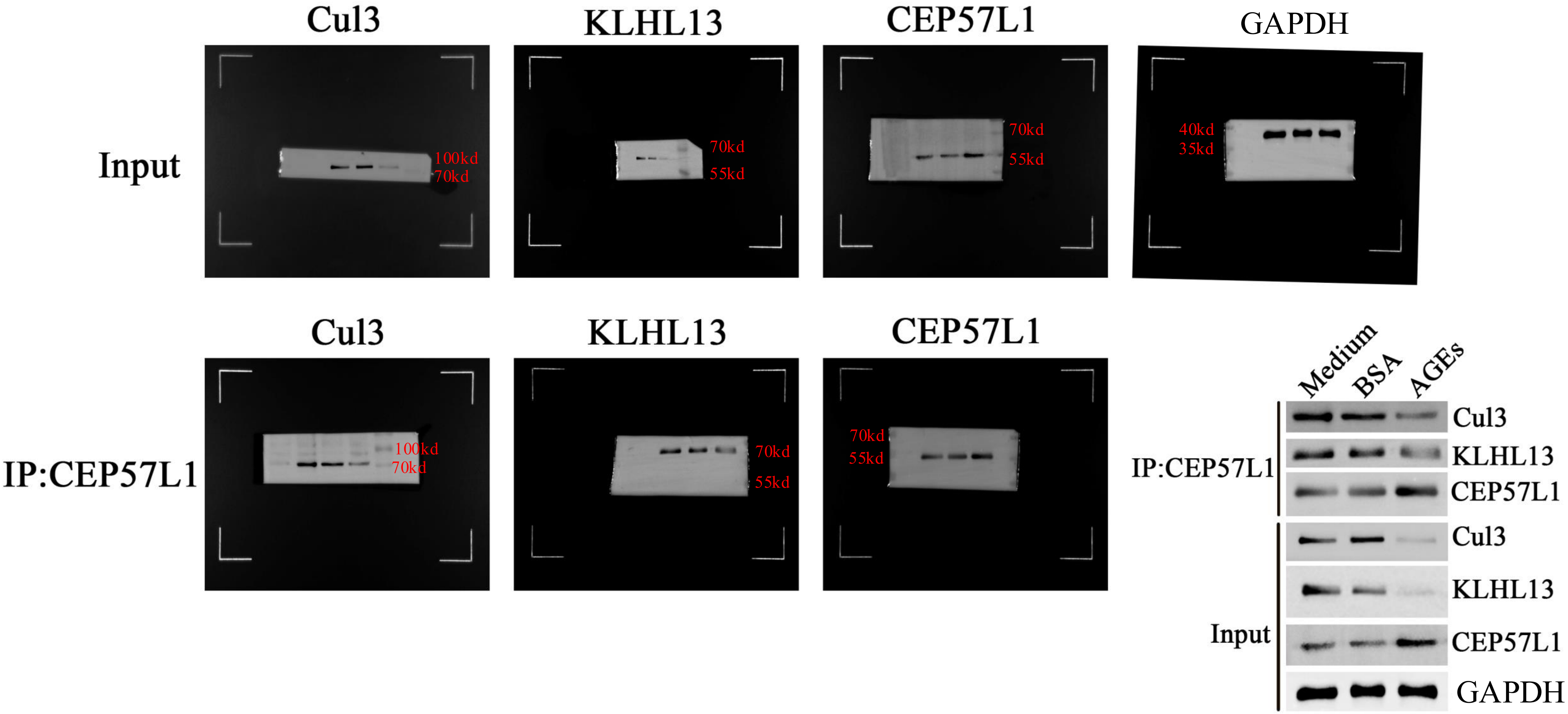

Cul3
KLHL13
CEP57L1
GAPDH
Input
100kd
70kd
70kd
55kd
70kd
55kd
40kd
35kd
Cul3
KLHL13
CEP57L1
IP:CEP57L1
100kd
70kd
70kd
55kd
70kd
55kd
Medium
BSA
AGEs
IP:CEP57L1
Cul3
KLHL13
CEP57L1
Input
Cul3
KLHL13
CEP57L1
GAPDH

Figure 5D

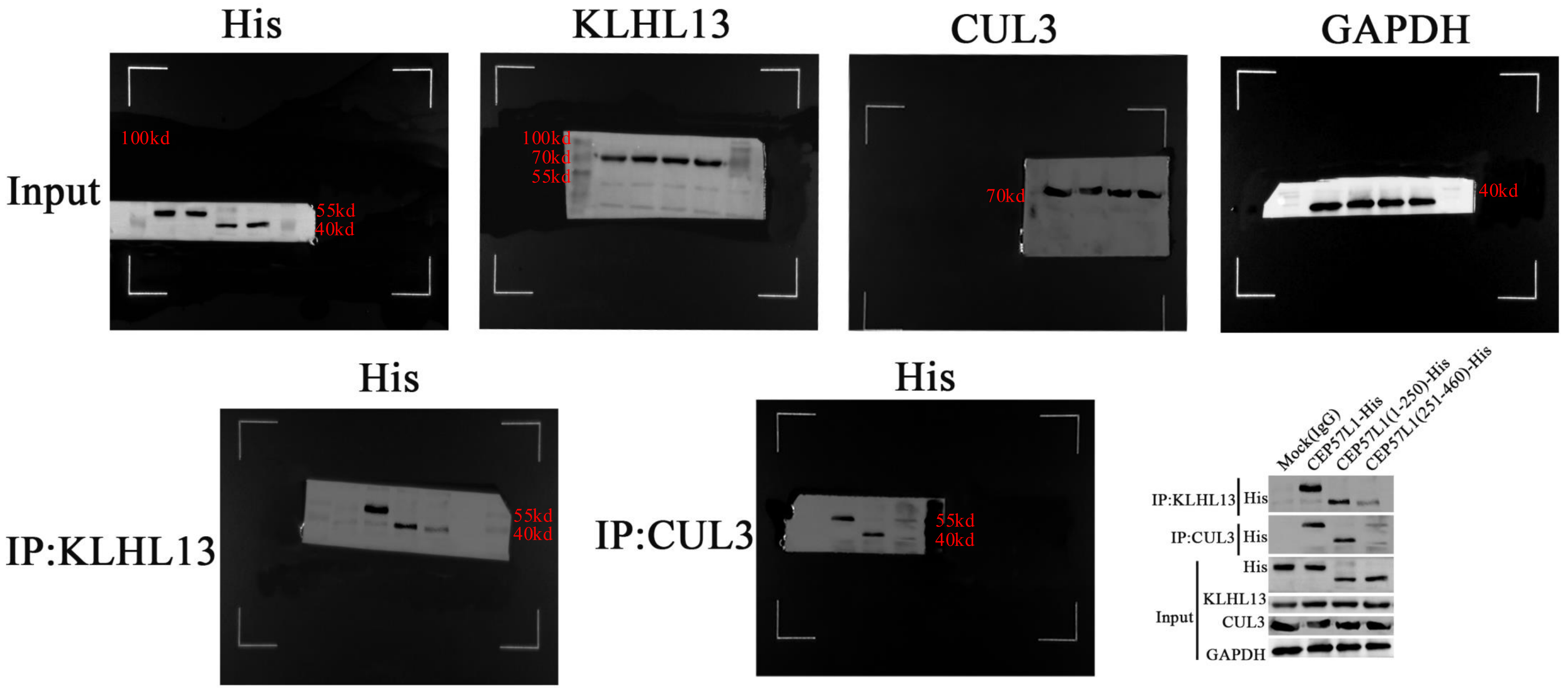

His
KLHL13
CUL3
GAPDH
Input
100kd
55kd
40kd
100kd
70kd
55kd
70kd
40kd
His
His
IP:KLHL13
55kd
40kd
IP:CUL3
55kd
40kd
Mock(IgG)
CEP57L1-His
CEP57L1(1-250)-His
CEP57L1(251-460)-His
IP:KLHL13
His
IP:CUL3
His
His
Input
KLHL13
CUL3
GAPDH

Figure 5E

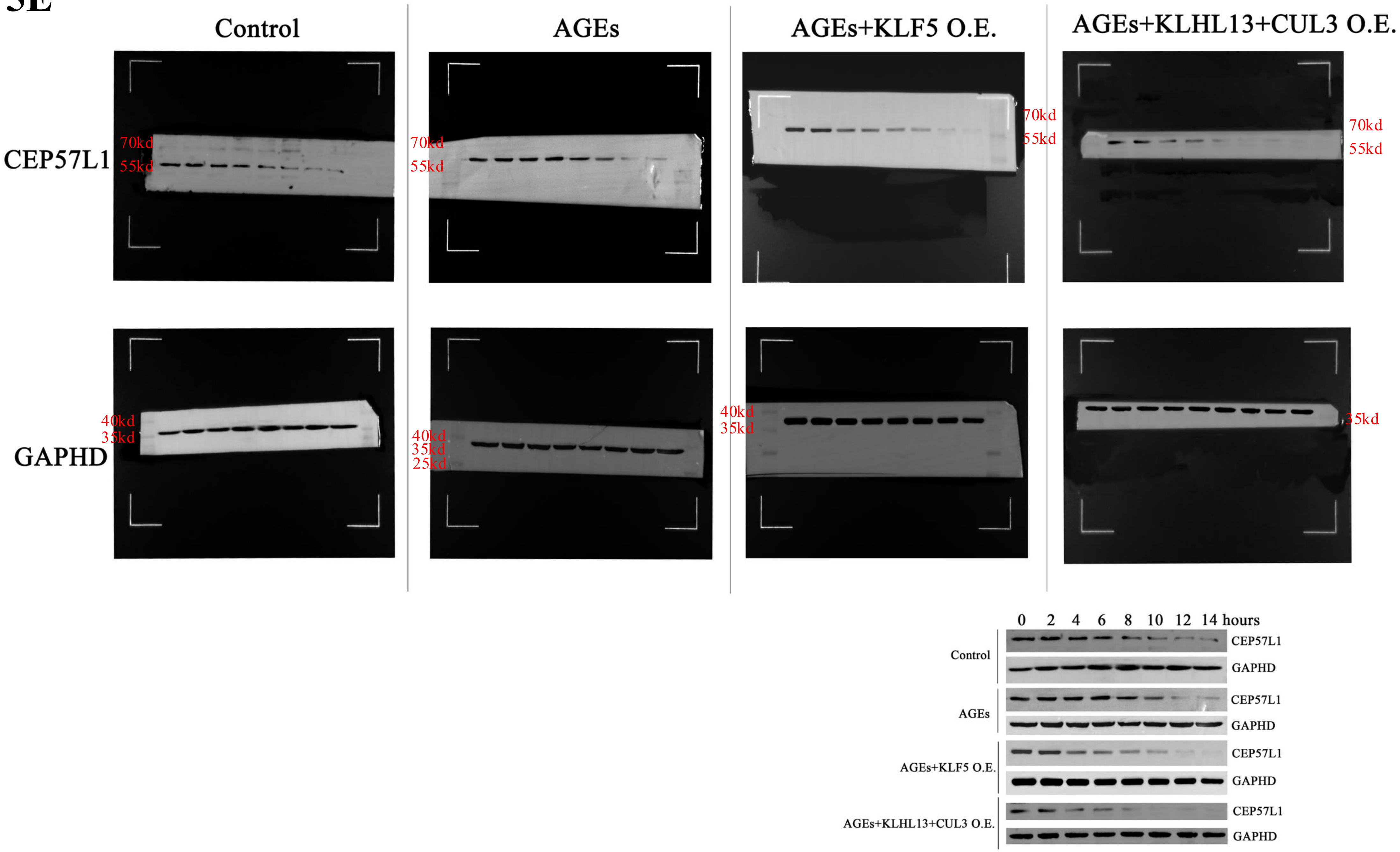

Control
AGEs
AGEs+KLF5 O.E.
AGEs+KLHL13+CUL3 O.E.
CEP57L1
GAPHD
70kd
55kd
40kd
35kd
25kd
0 2 4 6 8 10 12 14 hours
Control
AGEs
AGEs+KLF5 O.E.
AGEs+KLHL13+CUL3 O.E.
CEP57L1
GAPHD

Figure 5F

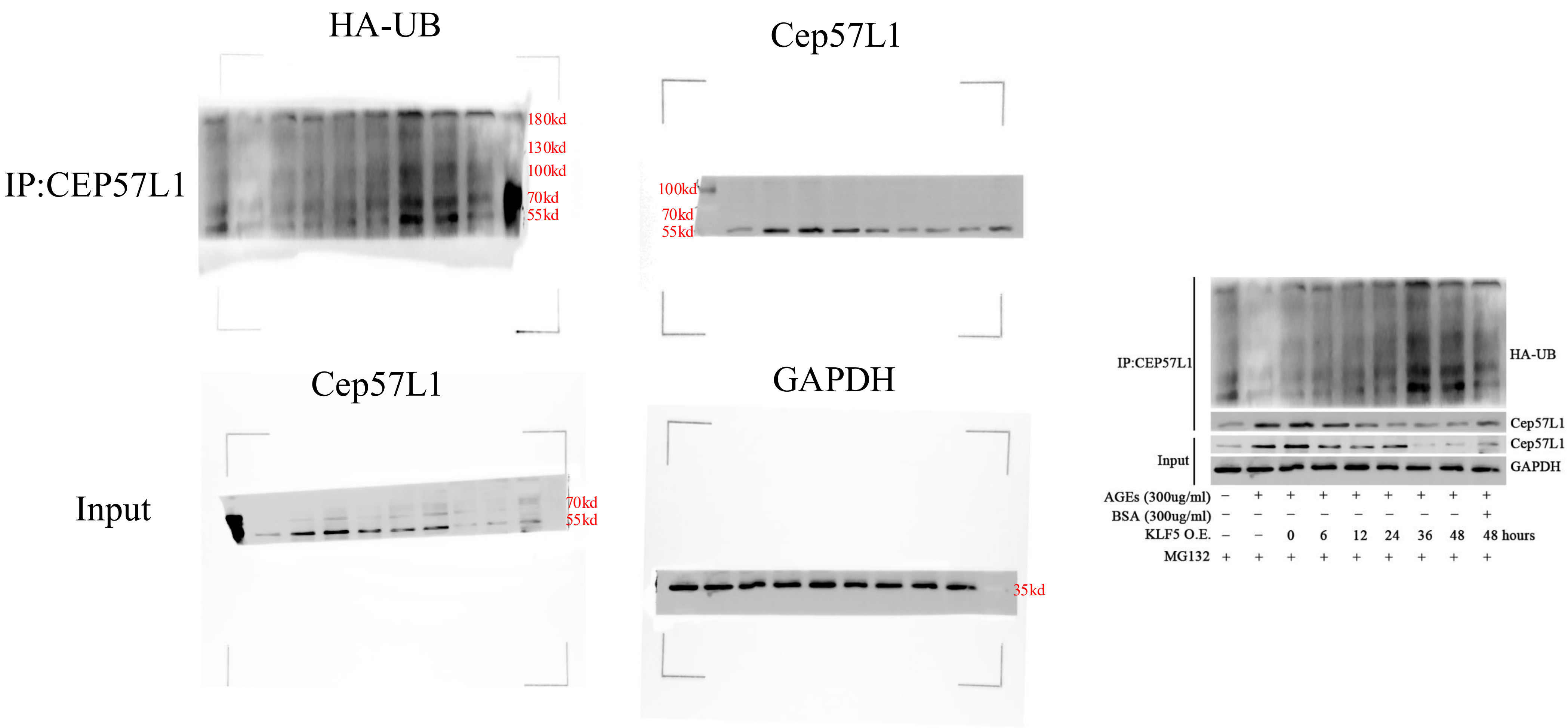
HA-UB
IP:CEP57L1
180kd
130kd
100kd
70kd
55kd
Cep57L1
100kd
70kd
55kd
Cep57L1
Input
70kd
55kd
GAPDH
35kd
IP:CEP57L1
HA-UB
Cep57L1
Input
Cep57L1
GAPDH
AGEs (300ug/ml) – + + + + + + + +
BSA (300ug/ml) – – – – – – – – +
KLF5 O.E. – – 0 6 12 24 36 48 48 hours
MG132 + + + + + + + + +

Figure 5G

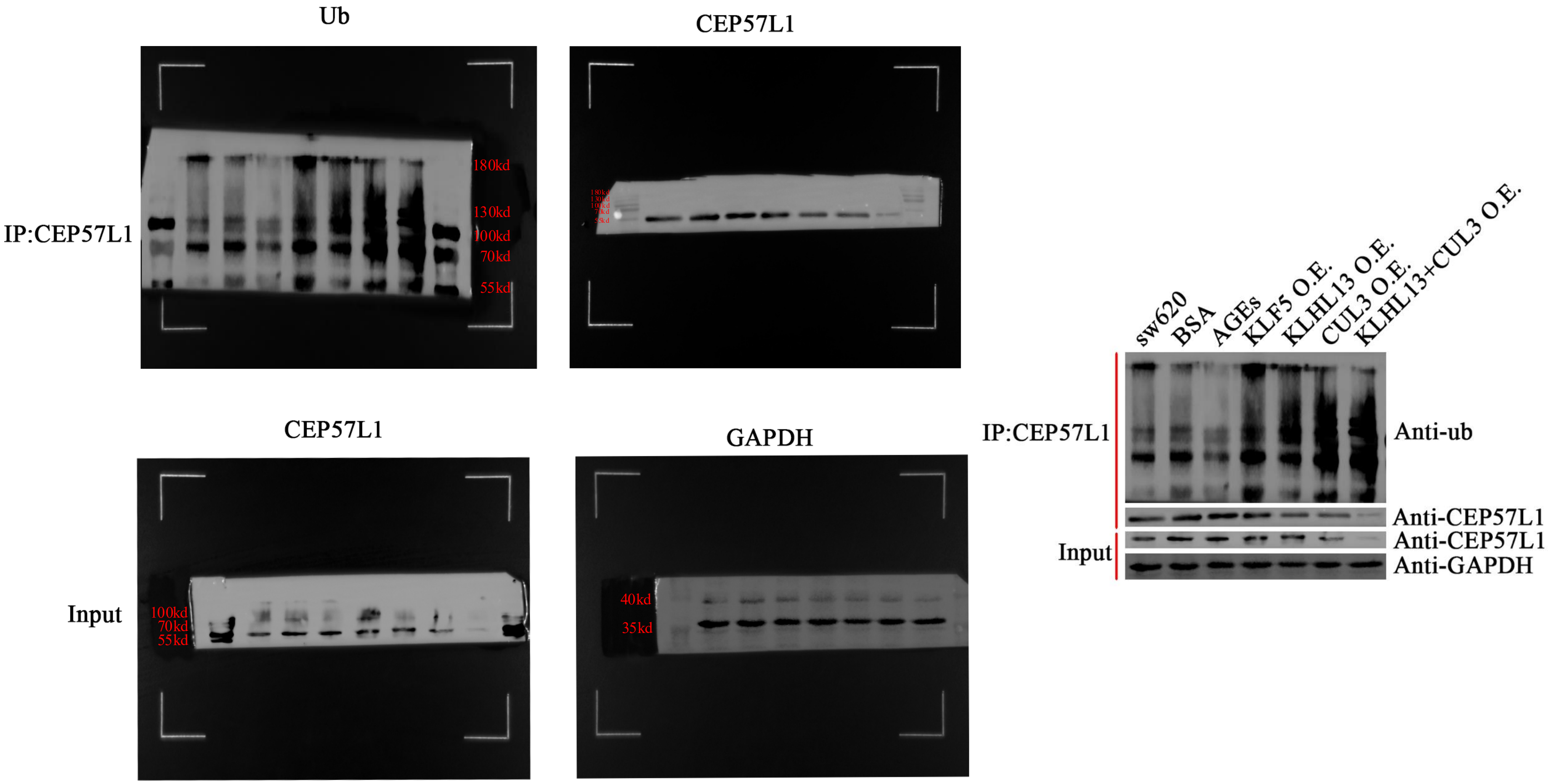
Ub
CEP57L1
IP:CEP57L1
180kd
130kd
100kd
70kd
55kd
CEP57L1
GAPDH
Input
100kd
70kd
55kd
40kd
35kd
sw620
BSA
AGEs
KLF5 O.E.
KLHL13 O.E.
CUL3 O.E.
KLHL13+CUL3 O.E.
IP:CEP57L1
Anti-ub
Anti-CEP57L1
Input
Anti-CEP57L1
Anti-GAPDH

Figure 5H

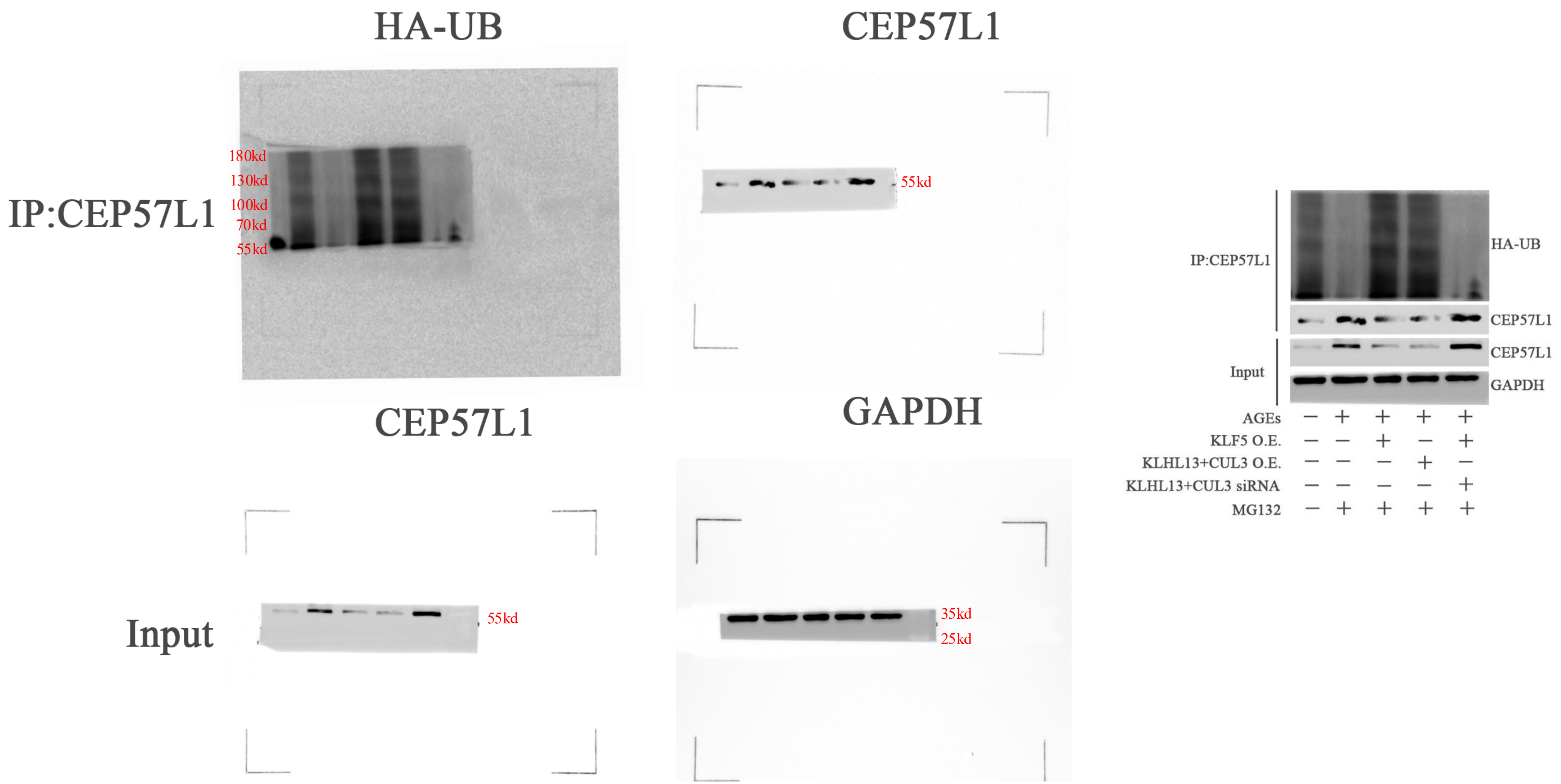
HA-UB
CEP57L1
IP:CEP57L1
180kd
130kd
100kd
70kd
55kd
55kd
CEP57L1
GAPDH
Input
55kd
35kd
25kd
IP:CEP57L1
HA-UB
CEP57L1
CEP57L1
Input
GAPDH
AGEs − + + + +
KLF5 O.E. − − + − +
KLHL13+CUL3 O.E. − − − + −
KLHL13+CUL3 siRNA − − − − +
MG132 − + + + +

Figure 7C

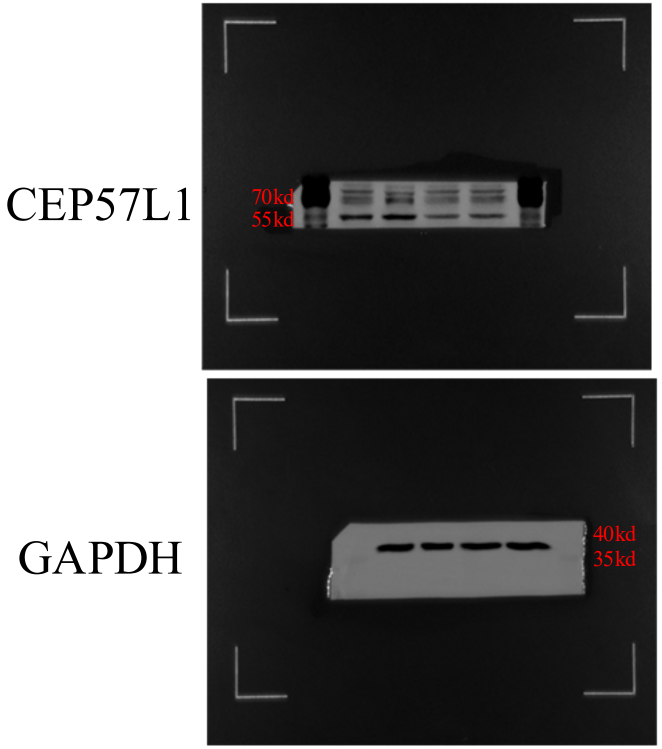
CEP57L1
70kd
55kd
GAPDH
40kd
35kd

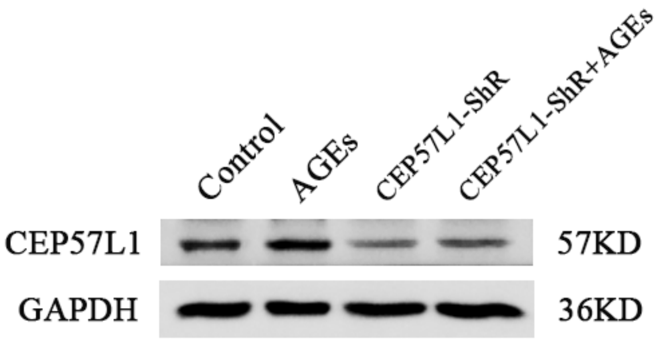
Control
AGEs
CEP57L1-ShR
CEP57L1-ShR+AGEs
CEP57L1
57KD
GAPDH
36KD
